# H3K27 Acetylation-driven IGF2BP2 Mutates during the Aging of MSCs, thereby Influencing Osteogenic Differentiation and Bone Aging

**DOI:** 10.7150/ijbs.122708

**Published:** 2026-01-01

**Authors:** Zimo Zhou, Kai Kang, Heran Wang, Boya Wen, Da Liu

**Affiliations:** 1 Department of Orthopedics, Shengjing Hospital of China Medical University, Shenyang, Liaoning, China.; 2 Department of Ultrasound, Shengjing Hospital of China Medical University, Shenyang, Liaoning, China.; 3 Hengyang Medical College, University of South China, Hengyang, Hunan, China.

**Keywords:** H3K27ac, IGF2BP2, mutation, osteogenic differentiation, bone aging, MSC senescence

## Abstract

Aging-related bone loss is closely linked to mesenchymal stem cell (MSC) senescence, but the underlying epigenetic mechanisms remain unclear. Here, the role of histone H3 lysine 27 acetylation (H3K27ac) and its downstream target IGF2BP2 in MSC aging are investigated. Integrated ChIP-seq and RNA-seq analyses revealed diminished H3K27ac levels in aged murine bone marrow-MSCs (BM-MSCs), accompanied by reduced IGF2BP2 expression. Functional studies demonstrated that both knockdown and overexpression of IGF2BP2 mitigated senescence phenotypes in hydrogen peroxide- and etoposide-induced models. The mutation frequency of H65Q, a key point mutation in IGF2BP2, exhibited variations according to age and sex, and enhanced its binding to *Hmga1* mRNA, stabilizing HMGA1 and activating the p53/p21 pathway to accelerate senescence. HMGA1 interacted with p53 to modulate DNA damage responses. Pharmacological inhibition of IGF2BP2 using CWI1-2 alleviated MSC senescence *in vitro* and enhanced bone regeneration in aged mice by improving bone mineral density and trabecular microstructure. These findings establish the H3K27ac-IGF2BP2-HMGA1 axis as a central regulator of bone aging and propose CWI1-2 as a promising therapeutic agent for age-related osteoporosis.

## Introduction

Aging is a multifactorial process marked by the accumulation of senescent cells and a progressive functional decline across tissues. The buildup of senescent cells—particularly mesenchymal stem cells (MSCs)—drives organismal aging, because of diminished proliferative capacity, impaired self-renewal, and aberrant differentiation potential. These impairments disrupt cellular renewal processes essential for tissue maintenance 1, 2. MSCs, pivotal for bone homeostasis, exhibit impaired osteogenic differentiation and self-renewal during aging, contributing to osteoporosis and fracture risk 3-5. Thus, elucidating the mechanisms underlying MSC aging and developing strategies to rejuvenate their function are of critical importance.

The progression of aging is influenced by diverse factors, among which epigenetic regulation serves as a major biological information storage system. Over time, repeated cycles of cell division, damage, and repair lead to gradual erosion of epigenetic information, which is considered a significant driver of senescence 6. Chemical modifications of histones, including acetylation, methylation, crotonylation, among others, constitute a vital component of epigenetics 7-9. During aging, the dynamics of these modifications are disrupted, compromising the precise regulation of gene expression and thereby promoting cellular senescence 10. In particular, acetylated histone H3 at lysine 27 (H3K27ac), an activation-associated mark, has attracted growing interest. Investigating age-dependent alterations in H3K27ac levels and their impact on downstream targets offers valuable insights into the epigenetic basis of aging.

Using integrated ChIP-seq and transcriptomic profiling of young versus aged murine bone marrow-MSCs (BM-MSCs), we mapped H3K27ac modification landscapes and identified IGF2BP2 as a key downstream target. Functional analyses revealed that IGF2BP2 modulates MSC aging and osteogenic differentiation through a DNA point mutation rather than expression-level changes. Mechanistically, this mutation regulates cellular senescence via the HMGA1/p53 pathway. Furthermore, pharmacological inhibition of IGF2BP2 using covalent binder CWI1-2 attenuated MSC aging, restored osteogenic potential *in vitro*, and enhanced bone regeneration *in vivo*. These findings uncover a multi-layered regulatory mechanism by which H3K27ac-driven IGF2BP2 contributes to MSC aging and highlight its therapeutic potential for treating age-related bone disorders.

## Materials and Methods

### Ethical statement and animal models

All animal experiments complied with the ethical guidelines published by the International Council for Laboratory Animal Science (ICLAS), ARRIVE guidelines and carried out in accordance with the U.K. Animals (Scientific Procedures) Act, 1986 and associated guidelines, EU Directive 2010/63/EU for animal experiments. The protocols were approved by the Ethics Committee of Shengjing Hospital, China Medical University (Approval Nos. 2024PS012K and 2024PS1661K). Male C57BL/6J mice were obtained from Beijing Huafukang Biotechnology Co., Ltd. The animals were housed in specific pathogen-free laboratory facilities until they reached age for subsequent experimental procedures. For natural aging studies, 6-, 15- and 24-month-old male C57BL/6J mice (n = 6 per group) were housed until they reached age for subsequent experimental procedures.

#### Pharmacological treatment

15-month-old male C57BL/6 mice were randomly assigned to control or CWI1-2 treatment groups (n = 6 per group). The CWI1-2 group received intraperitoneal injections of CWI1-2 (MedChemExpress, MCE, USA) at 5 mg/kg every other day; control animals received an equivalent volume of vehicle. After two months, mice were euthanized for subsequent analysis.

#### Euthanasia and tissue harvest

Mice were euthanized via carbon dioxide inhalation. Femurs were dissected and fixed in 4% paraformaldehyde for further processing.

#### Micro-computed tomography (Micro-CT)

Fixed femoral samples were scanned using a micro-CT system (VNC-102, PINGSENG, China). A standardized region of interest (ROI) was selected 1.0 mm distal to the growth plate of the femoral head. Images were thresholded at a value of 1151 to distinguish cortical and trabecular bone. Morphometric parameters—including bone volume fraction (BV/TV), trabecular number (Tb.N), trabecular separation (Tb.Sp), and bone mineral density (BMD)—were calculated from three repeated scans per ROI and analyzed using augmented reality-assisted tools.

### Cell culture, osteogenic differentiation and CWI1-2 treatment

The C3H/10T1/2 cell line (Procell, cat. no. CL-0325, China) was cultured in C3H/10T1/2 Cell-Specific Culture Medium (Procell) under 5% CO_2_ at 37 ℃.

Primary bone marrow-derived mesenchymal stem cells (BM-MSCs) were isolated from young (8-week-old) and aged (15-month-old) C57BL/6J mice by flushing femoral marrow cavities and maintained in minimum essential medium (MEM)-α (Gibco, USA) supplemented with 15% fetal bovine serum (Procell).

For osteogenic differentiation, C3H/10T1/2 cells were induced with Dulbecco's modified Eagle's medium/F12 (DMEM/F12) (Gibco) medium containing 10 mM β-glycerophosphate (Sigma-Aldrich, USA), 50 μg/mL ascorbic acid (GLPBIO, USA), and 100 nM dexamethasone (Beyotime, China). For drug treatment, cells were exposed to 1 μM CWI1-2 or vehicle for 48 hours.

### Senescence models

Hydrogen peroxide-induced senescence was achieved by treating cells with 200 μM H_2_O_2_ (Macklin, China) in MEM-α for 2 hours, followed by recovery in complete medium for 24 hours 11.

Etoposide powder (Beyotime) was prepared into 10 mM storage solution using DMSO (Solarbio, China). For etoposide-induced senescence, cells were treated with 5 μM working fluid configuration for 48 hours 12.

### Chromatin immunoprecipitation sequencing (ChIP-seq) and RNA sequencing

#### ChIP-seq library construction and data analysis

For ChIP-seq, ≥ 2×10^7^ cells per group were processed using an anti-H3K27ac antibody (Table S1)^13^. Libraries were constructed and sequenced on the MGISEQ-T7 platform (Seqhealth Technology, China). A paired-end library (~300bp) was prepared specifically for this purpose, followed by rigorous quality control of the generated sequencing data. Subsequently, bioinformatics analysis was employed to analyze the ChIP-seq data. Data analysis was supported by Seqhealth Technology Co., LTD.

#### RNA-seq library construction

For RNA-seq, ≥ 1×10^7^ cells were used. Data processing and bioinformatic analyses were supported by Seqhealth Technology Co., Ltd.

### RIP-seq data analysis

The RIP-seq dataset is murine-derived, and has been published (DOI:10.1002/jcp.30919) 14, which can be accessed from BIG Data Center of China National Center for Bioinformation, under the accession number: PRJCA024623. The visual analysis was conducted using TBTools-II software (version 2.092) 15. The sequences were visually analyzed using Integrative Genomics Viewer (Version 2.17.4).

### Bioinformatics analysis

Single-cell dataset GSE169396 (Platform: GPL11154) 16 and validation set GSE226325 (Platform: GPL24676) were acquired from GEO database and analyzed via the ASSISTANT for Clinical Bioinformatics platform and GEO2R, respectively.

### Western blotting

Cells were lysed in RIPA buffer (Epizyme, China) containing protease and phosphatase inhibitors (Epizyme). Proteins were separated by SDS-PAGE, transferred to PVDF membranes (Millipore, USA). The membranes were incubated with protein-free rapid blocking buffer (Epizyme) at RT for 40 minutes. Incubation with primary antibodies was carried out overnight while continuously stirring at 4 °C followed by HRP-conjugated secondary antibodies for another 90 minutes at RT (Table S1). Signals were detected using a chemiluminescence kit (Epizyme) and imaged (Amersham Imager 680, GE, USA).

### Senescence β-galactosidase staining

Cellular senescence was assessed using a senescence β-galactosidase staining kit (Beyotime) according to the manufacturer's instructions.

### Real-time reverse transcription-polymerase chain reaction assay (RT-qPCR)

The total RNA was extracted using Trizol reagents, and reverse transcription was performed according to the manufacturer's recommendations using a Reverse Transcription Kit (Biosharp, China). Quantitative reverse transcription PCR was conducted on the ABI7500 rapid real-time PCR system (Applied Biosystems, USA) with SYBR Green qPCR Mix kit (Biosharp). The relative mRNA expression levels were normalized using 2^-△△CT^ method, with GAPDH serving as the endogenous control. All primers were listed in Table S2.

### RNA immunoprecipitation (RIP)

The Bersinbio^TM^ RIP kit (Bersinbio, China) was utilized following the manufacturer's instructions. In brief, at least 1×10^7^ cells were harvested and resuspended in polysome lysis buffer supplemented with RNase and protease inhibitors, respectively. For bone tissue, the tissue was first ground into a fine powder in liquid nitrogen, after which the aforementioned reagents were added. Genomic DNA was removed prior to the immunoprecipitation step, during which antibodies and Protein A/G magnetic beads were introduced. The immunoprecipitated RNA was then extracted using Trizol. Following purification, the RNA was subjected to reverse transcription, and the resulting cDNA was analyzed by RT-qPCR.

### Co-Immunoprecipitation (Co-IP)

A total of 1×10^7^ cells were harvested and lysed using IP lysis buffer (Epizyme) to collect the supernatant. Subsequently, specific antibodies were added, and the mixture was incubated overnight at 4℃. Protein A/G magnetic beads (MCE) were then introduced to capture the immune complexes, which were isolated via magnetic separation following incubation. The immune complexes were washed and resuspended in loading buffer. After heating in a water bath, the supernatant was obtained by centrifugation and subjected to further detection.

### MRNA stability measurement

Actinomycin D effectively inhibits total mRNA transcription 17. Following the cell processing, cells were treated with 5 μg/mL actinomycin D (GLPBIO). The mRNA level of *Hmga1* was quantified by RT-qPCR at various time points, and the relative expression was determined using the formula 2^-ΔCT^.

### Cell transfection

The small interfering RNA (siRNA) and plasmid overexpressing *Igf2bp2* were procured from OBiO Technology (China). Transfection was carried out following the manufacturer's protocol. In brief, siRNA (or plasmid) and the Lipofectamine 3000 transfection reagent (Invitrogen, USA) are first diluted to their respective working concentrations using Opti-MEM (Gibco). Subsequently, the two solutions are combined and incubated prior to being introduced to the cells. The sequences of *siR-Igf2bp2* and *siR-Hmga1* can be found in Table S3. The sequence information of the plasmid was shown in Figure S13, Figure S14.

### Immunofluorescence and RNA fluorescence in situ hybridization (FISH)

Cells were fixed with 4% paraformaldehyde for 30 minutes, subsequently permeabilized with 0.5% Triton X-100 at room temperature, and blocked with bovine serum albumin (BSA). The primary and secondary antibodies were applied sequentially to allow for specific antigen-antibody immunoreaction. Nuclei were stained with 0.5 μg/mL DAPI (4',6-diamidino-2-phenylindole; Solarbio). Fluorescent imaging and analysis were conducted using an Olympus BX51 microscope system.

The antibodies used are listed in Table S1.

For FISH analysis, cells were fixed using an in situ hybridization fixation solution (Servicebio, China) and subsequently treated with proteinase K (5 μg/mL) for digestion. Following incubation with pre-hybridization solution, the hybridization solution containing the probe was applied to perform hybridization. The probe sequence for *Hmga1* mRNA is presented in Table S4.

### Immunohistochemistry (IHC)

The femur sections from mice were antigen unmasked using citrate. Following quenching of endogenous peroxidase activity, the slices were blocked and incubated with the antibody. Subsequent steps were performed utilizing an immunoperoxidase assay kit (Servicebio). Quantitative analysis of positively stained areas was conducted using ImageJ software.

### Molecular subcellular co-localization analysis

Fluorescence images were analyzed using the Colocalization Finder plugin in ImageJ, and the Pearson's correlation coefficient was calculated for statistical analysis.

### Cell proliferation assay

The cell viability was assessed using the Cell Counting Kit-8 (CCK-8) assay (GLPBIO). Following the supplier's protocol, treated cells were seeded in 96-well plates and cultured under standard conditions for different times. At each time point, a volume of 10 μL CCK-8 reagent was added to each well, and the absorbance at 450 nm was measured using a flatbed instrument after incubation for 2 hours.

### Alkaline phosphatase staining and activity assay

The osteogenic differentiated cells were rinsed with PBS, fixed in 4% paraformaldehyde, and subsequently stained using a 5-bromo-4-chloro-3-indole-phosphate/nitroblue tetrazolium (BCIP/NBT) kit (Beyotime), following the manufacturer's instructions. ALP activity was assessed utilizing an ALP activity kit (Elabscience, China) as per the manufacturer's protocol.

### Alizarin red S staining and quantitative assay

Alizarin red S staining kit (Biosharp), following the manufacturer's protocol: fixation, staining with alizarin red S, and observation under a microscope. After drying, 10% cetylpyridine chloride aqueous solution (Solarbio) was added to dissolve the mineralized nodules bound by alizarin red. The resulting solution's optical density was measured at 562 nm using BioTek microplate reader.

### Protein structure analysis and protein-protein docking simulations

The protein sequence was retrieved from the National Center for Biotechnology Information (NCBI). The three-dimensional structure of the protein was obtained from SWISS-MODEL 18. Protein**-**Protein docking analysis was conducted using HawkDock 19, 20. The visualizations of protein structures were performed with PyMOL (Version 3.0.3).

### Sequence conservation analysis

The target sequences of IGF2BP2 from various species were retrieved from the NCBI database. Sequence comparison and visualization were conducted using MEGA software (Version 7.0.26), and the logo of sequences was generated via the WebLogo3 platform 21. Genome Aggregation Database (gnomAD) was used to analysis the probability of gene mutations in the population.

### Statistical analysis

The results of this study were analyzed by using GraphPad Prism 10 software. The data are expressed as the mean ± standard deviation (SD). Differences between different groups were analyzed by using unpaired two-tailed sided Student's t tests. P < 0.05 was regarded as significant.

## Results

### Age-associated alterations in H3K27ac modification in BM-MSCs

**Figure 1:**
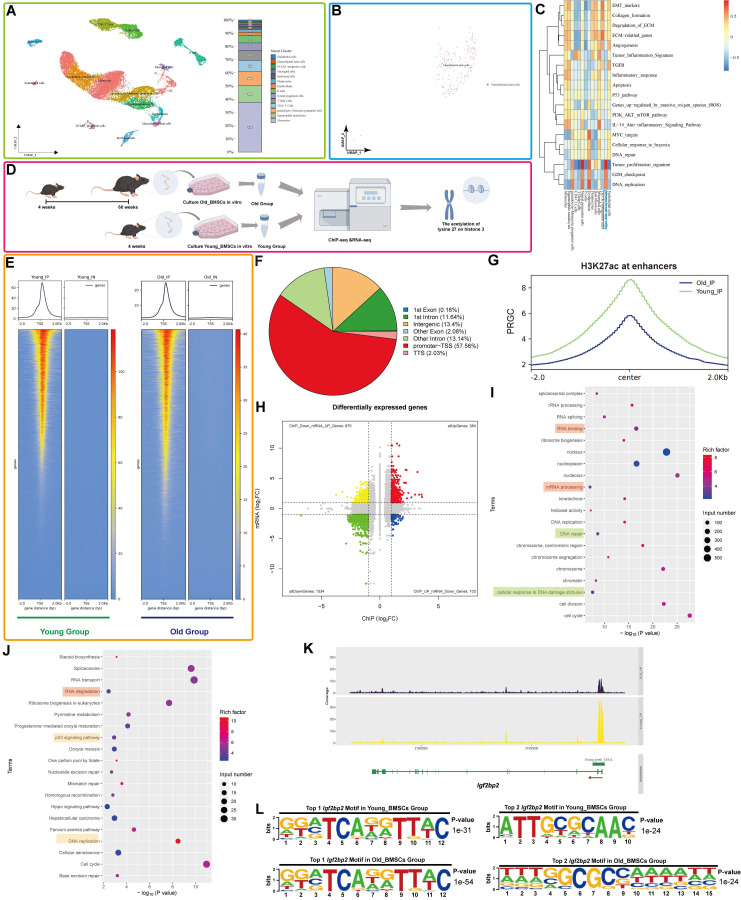
** Integrated analysis of H3K27ac ChIP-seq and RNA-seq reveals age-related alterations and H3K27ac-driven* Igf2bp2* change in mouse BM-MSCs.** (A) Single-cell UMAP projection of femoral head cells. Single-cell transcriptomes are subjected to dimensionality reduction and clustering via the UMAP algorithm, with subsequent detailed cell type annotation. Distinct colors represent heterogeneous cell clusters. The right panel displays the proportional composition of cell types, with the y-axis indicating percentage distribution. (B) UMAP projection specifically highlighting MSC clusters within the femoral head. (C) Pathway activity heatmap across cell subpopulations. Columns denote distinct cell subsets, while rows represent biological pathways. A color gradient from red (elevated activity) to blue (reduced activity) visualizes pathway enrichment scores. (D) Schematic workflow for integrative H3K27ac ChIP-seq and RNA-seq analysis of aging-associated changes in BM-MSCs (generated using Figdraw, version 2.3.0). (E) Distribution of H3K27ac reads relative to TSS of peak-annotated genes. (F) Differential distribution of H3K27ac peaks between the Young Group and the Old Group. (G) Enhancer-associated H3K27ac signal distribution across aging groups. The x-axis ("Center") denotes midpoint positions of 6-kb genomic bins; y-axis represents reads per genome coverage (RPGC). (H) Integrative ChIP-seq/RNA-seq volcano plot. X-axis: H3K27ac ChIP-seq signal intensity; y-axis: RNA-seq log2 fold change. Dashed lines indicate significance thresholds. (I, J) GO and KEGG enrichment analyses of downregulated DEGs. (K) H3K27ac ChIP-seq read density across the *Igf2bp2* locus in Young versus Old groups. (L) Top two H3K27ac-binding motifs at* Igf2bp2* loci in Young and Old groups. The results are shown as means ± SD, n ≥ 3 independent experiments; two-tailed Student's *t*-test.

Single-cell clustering analysis of human femoral head tissue identified distinct cellular populations, with mesenchymal stem cells (MSCs) accounting for approximately 1% of all cells (Figure 1A). Further subclustering and visualization confirmed the identity of the MSC population (Figure 1B). Gene Set Variation Analysis (GSVA) revealed significant enrichment of DNA-related pathways in MSCs (Figure 1C).

Comparison of RNA-seq data from BM-MSCs of aged (15-month-old) and young (8-week-old) mice showed elevated expression of senescence markers (*Trp53*, *Cdkn1a* (*p21*), *Cdkn2a* (*p16*)) and SASP factors (*Il-6*, *Cxcl1* and *hmgb1*), alongside reduced expression of proliferation and nuclear integrity genes (*Pcna*, *Lmnb1*) (Figure S1), indicating enhanced senescence in aged BM-MSCs.

Integrated analysis of H3K27ac-ChIP-seq and RNA-seq was performed to identify genes linked to both MSC aging and H3K27ac modification (Figure 1D). ChIP-seq profiling revealed a global reduction in H3K27ac signal in aged BM-MSCs, particularly at enhancer regions (Figure 1F-G, S2A). Although promoter-proximal H3K27ac peaks were predominant in both groups, a marked decrease in total reads was observed in Old BM-MSCs (Figure 1E), consistent with age-related erosion of epigenetic landscapes. Correlation analysis of the genes identified from the IP showed a strong correlation between the Young and Old groups (R=0.93), suggesting that the IP results are of good technical quality and are suitable for further analysis. Enhancer H3K27ac occupancy levels are illustrated in Figure S2B, C.

RNA-seq analysis demonstrated clear inter-group differential expression, with minimal intra-group variation (Figure S2D-E). A volcano plot identified numerous differentially expressed genes (DEGs) (Figure S2F). Functional enrichment analysis revealed that these DEGs were associated with nuclear and chromatin-related biological processes (Gene Ontology (GO) terms) and pathways related to cellular lifespan and DNA metabolism (Kyoto Encyclopedia of Genes and Genomes (KEGG)) (Figure S2G, H).

### IGF2BP2 expression correlates with H3K27ac changes in aging BM-MSCs

Integration of ChIP-seq and RNA-seq datasets identified genes exhibiting concurrent reduction in both H3K27ac enrichment and expression with aging (|Log2(fold change)| ≥ 1 and p-value < 0.05) (Figure 1H). In light of the diminished H3K27ac modification levels observed in the Old Group, we identified genes with reduced read depth in ChIP-seq and decreased expression in RNA-seq as targets for GO and KEGG analyses. Functional annotation highlighted involvement in DNA/RNA-related processes and cellular aging pathways (Figure 1I, J).

Focusing on *Igf2bp2*, a key RNA modification-related gene, we observed a significant age-dependent decline in H3K27ac enrichment at its transcription start site (TSS) and across the entire genomic locus in aged BM-MSCs (Figure 1K, S3A). Motif analysis identified two of the most relevant conserved *Igf2bp2*-binding motifs in different groups, with sequence logos diverging between age groups (Figure 1L).

Consistent with epigenetic changes, RNA-seq revealed decreased IGF2BP2 expression in aged BM-MSCs, a trend corroborated in an independent validation dataset (GSE226325) (Figure S2I). We also analyzed and visualized the RNA levels of histone acetyltransferase *Ep300* and *Igf2bp2* in Single-cell clustering 22. The expression levels of *Ep300* and *Igf2bp2* were significantly higher in MSCs compared to other cell types (Figure S3B, C). This result indicates that, in comparison to other cell types, the regulation of H3K27ac modification and the influence of IGF2BP2 on MSCs are more pronounced and robust.

### Reduced H3K27ac and IGF2BP2 levels in senescent MSCs

**Figure 2:**
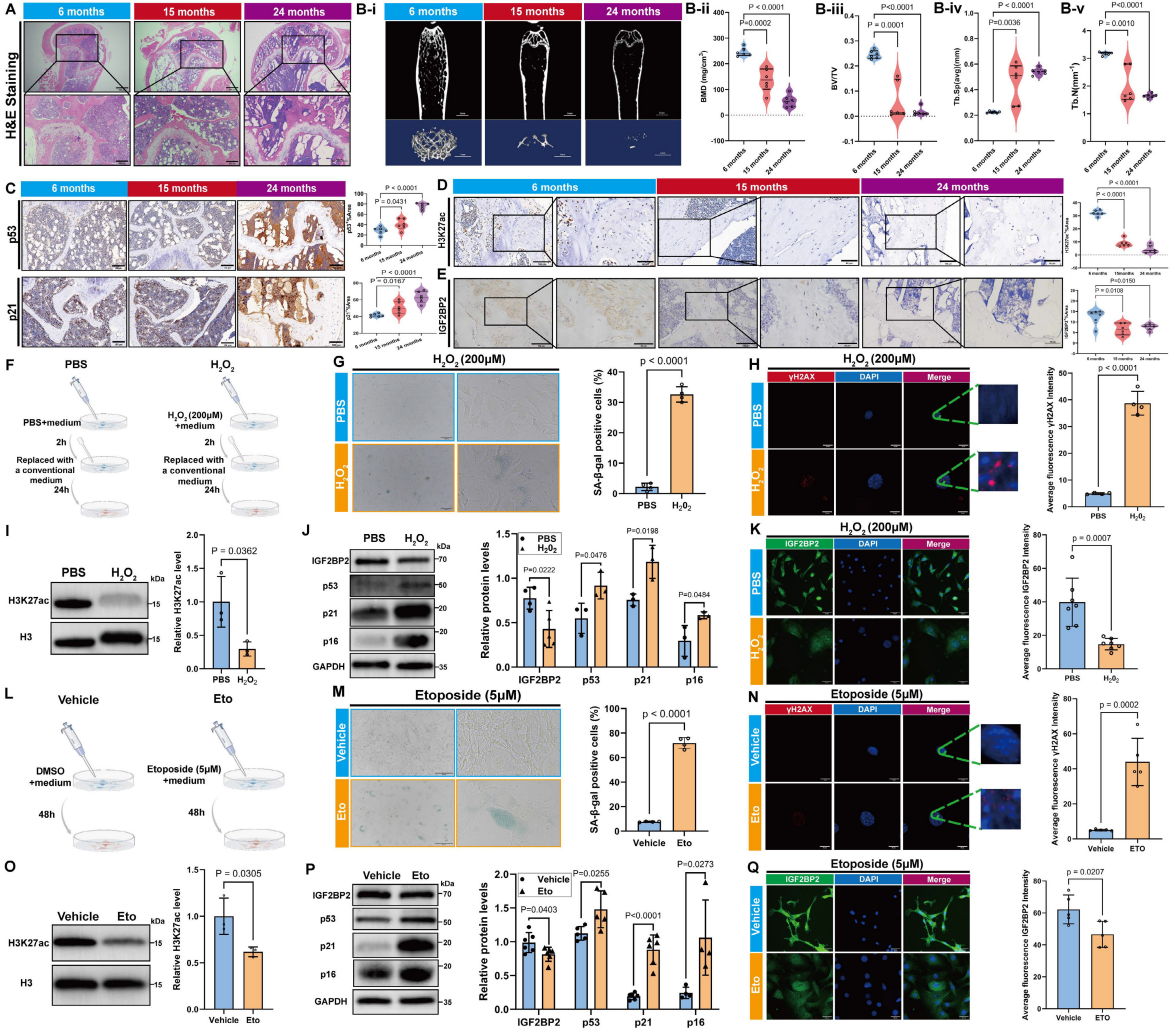
** Age-associated alterations in H3K27ac and IGF2BP2 expression across *in vivo* and *in vitro* models.** (A) H&E-stained bone tissue sections from age-stratified mice (scale bars: 500 μm main, 200 μm trabecular insets). (Bi-v) Micro-CT quantitation of bone parameters: BMD, BV/TV, Tb.Sp, and Tb.N. (C) IHC detection of p53 and p21 in bone sections (scale bar: 100 μm). (D, E) IHC analysis of H3K27ac and IGF2BP2 protein expression (scale bars: 100 μm main, 50 μm insets). (F, L) Senescence induction via 200 μM H_2_O_2_ or 5 μM etoposide (Figdraw-generated schematics). (G, M) SA-β-Gal staining (scale bars: 2000 μm). Green-stained cells denote SA-β-Gal (+) populations; quantified positivity rates are shown. (H, N) IF detection of γH2AX foci (red: γH2AX; blue: DAPI; scale bar: 50 μm). Fluorescence intensity was quantified. (I, O) Western blot and densitometric analysis of H3K27ac (normalized to histone H3). (J, P) Western blot analysis of IGF2BP2 and senescence markers (normalized to GAPDH). (K, Q) IF mapping of IGF2BP2 in H_2_O_2_ or etoposide-treated cells (scale bar: 500 μm). The results are shown as means ± SD, n ≥ 3 independent experiments; two-tailed Student's *t*-test.

Aged mice (15- and 24-month-old) displayed increased bone marrow adiposity, loss of trabecular bone and thinning of cortical bone (Figure 2A, Bi-v), elevated expression of senescence markers (p53, p21, and p16), and decreased levels of osteogenic markers (RUNX2, Osterix, and OCN) (Figure 2C, S4). Consistent with these changes, both H3K27ac modification levels and IGF2BP2 expression—at mRNA and protein levels—were reduced in bone tissue from aged mice (Figure 2D, E, Figure S4E).

Bone structural integrity relies on the cortical shell and the metaphyseal trabecular network, which collectively provide mechanical strength and facilitate physiological functions 23. Age-related decline in bone mineral density and disorganization of collagen fibers contribute to increased bone fragility and fracture risk 24. Concurrently, cortical bone undergoes thinning due to imbalanced periosteal deposition and endosteal resorption, while trabecular architecture becomes progressively compromised 23. Thus, assessment of cortical thickness and trabecular parameters via micro-CT imaging is essential for evaluating osteoporotic progression 25. In line with standard guidelines, we selected specific micro-CT parameters that robustly reflect age-related bone loss, cortical thinning, and trabecular deterioration.

As age increases and aging accumulates, the level of the transcription factor p53 continues to rise. The upregulation and sustained activation of p53 directly regulate the transcription of p21, leading to an increase and persistent accumulation of p21 and p16 in aging tissues and cells, thereby making it a reliable biomarker of cellular aging 26. RUNX2, Osterix and OCN are key transcription factors involved in osteoblast differentiation and play crucial roles in bone formation. The signaling pathways and molecular targets they regulate have been well established as significant downstream indicators of osteogenic differentiation 27-29. Numerous studies have demonstrated that RUNX2, Osterix and OCN are markedly upregulated during osteogenic differentiation, supporting their use as effective markers of this process 30.

To validate these findings *in vitro*, we established models of senescence in C3H/10T1/2 Clone 8 cells using hydrogen peroxide (H_2_O_2_) and etoposide treatment (Figure 2F, I). H_2_O_2_-induced senescence was confirmed by an increased proportion of SA-β-gal-positive cells (Figure 2G), elevated γH2AX fluorescence (Figure 2H), and upregulation of p53, p21 and p16 (Figure 2J). Concomitantly, H3K27ac and IGF2BP2 levels were significantly downregulated (Figure 2I-K). Similar results were observed in etoposide-induced senescence, underscoring the consistent suppression of the H3K27ac-IGF2BP2 axis across different senescence models (Figure 2M-Q).

### Modulation of IGF2BP2 expression attenuates MSC senescence

We designed a specific small interfering RNA (*siR-Igf2bp2*) and an overexpression plasmid (*OE-Igf2bp2*) to manipulate *Igf2bp2* expression. Transfection with *siR-Igf2bp2* or *OE-Igf2bp2* did not affect senescence under normal conditions, as indicated by unchanged SA-β-gal staining (Figure S5), but significantly altered *Igf2bp2* mRNA and protein levels (Figure S6).

**Figure 3:**
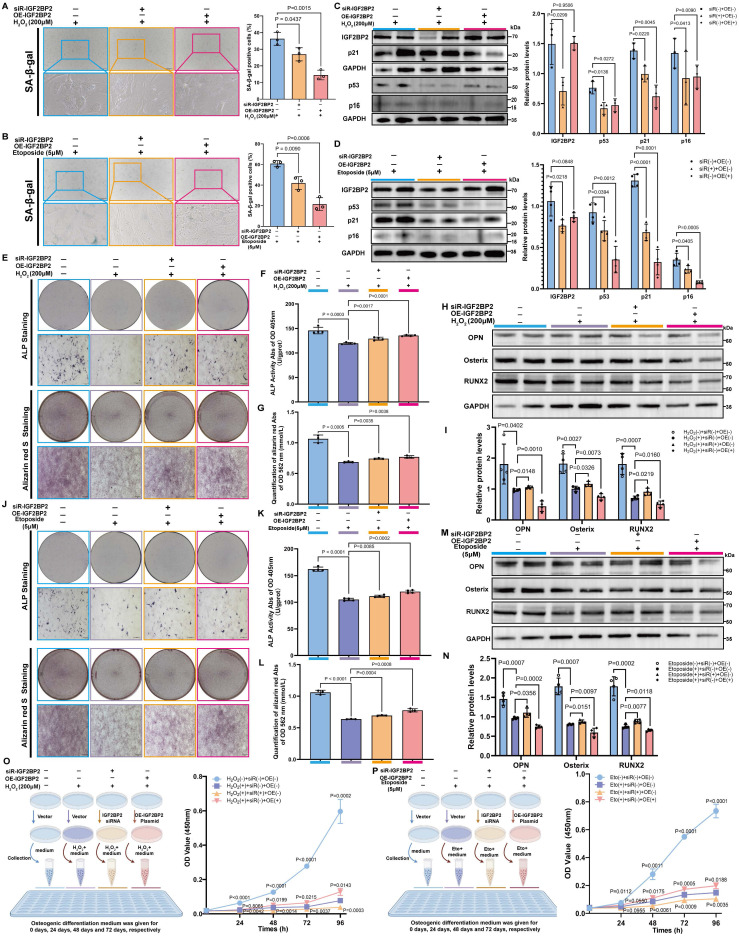
** The Impact of IGF2BP2 on senescent MSC phenotypes and osteogenic differentiation.** (A, B) SA-β-Gal staining of senescent MSCs (scale bars: 2000 μm). (C, D) Western blot and densitometry of IGF2BP2 and senescence markers (normalized to GAPDH). (E, J) ALP and ARS staining (scale bars: 2000 μm). Insets show magnified mineralized nodules. (F, K) Quantified ALP activity. (G, L) ARS absorbance quantification. (H, I, M, N) Western blot and densitometry of osteogenic markers (normalized to GAPDH). (O, P) CCK-8 proliferation assay. Left: Experimental schematic; right: time-course absorbance data (purple: age-induced control). The results are shown as means ± SD, n ≥ 3 independent experiments; two-tailed Student's *t*-test.

Both knockdown and overexpression of* Igf2bp2* significantly alleviated senescence in H₂O₂- and etoposide-treated MSCs, reflected by reduced SA-β-gal positivity (Figure 3A, B). Western blot analysis confirmed efficient *Igf2bp2* knockdown in both models, while its overexpression did not markedly alter baseline levels. Strikingly, p53, p21 and p16 protein levels were downregulated in both intervention groups (Figure 3C, D). Reduced γH2AX intensity further supported attenuated DNA damage response (Figure S7). These results suggest that altering *Igf2bp2* expression does not provoke senescence under normal conditions but significantly counteracts stress-induced senescence.

### *Igf2bp2* modulation restores osteogenic potential in senescent MSCs

Senescent MSCs showed compromised osteogenic capacity, with decreased ALP activity, reduced mineralization, and downregulation of osteogenic markers. Both knockdown and overexpression of *Igf2bp2* partially rescued these impairments, with similar restorative effects observed in H₂O₂- and etoposide-induced models (Figure 3E-M).

Notably, osteogenic markers were significantly suppressed in senescence-induced groups but were partially recovered following* Igf2bp2* knockdown. In contrast, overexpression further reduced their expression below the levels in senescence-induced groups (Figure 3H, I, M, N). Assessment of cell proliferation during osteogenic induction revealed that senescence significantly suppressed proliferation. Interestingly, proliferation was further inhibited in the knockdown group but was markedly restored by* Igf2bp2* overexpression (Figure 3O, P).

### IGF2BP2 stabilizes *Hmga1* mRNA and promotes HMGA1 expression in MSCs

**Figure 4:**
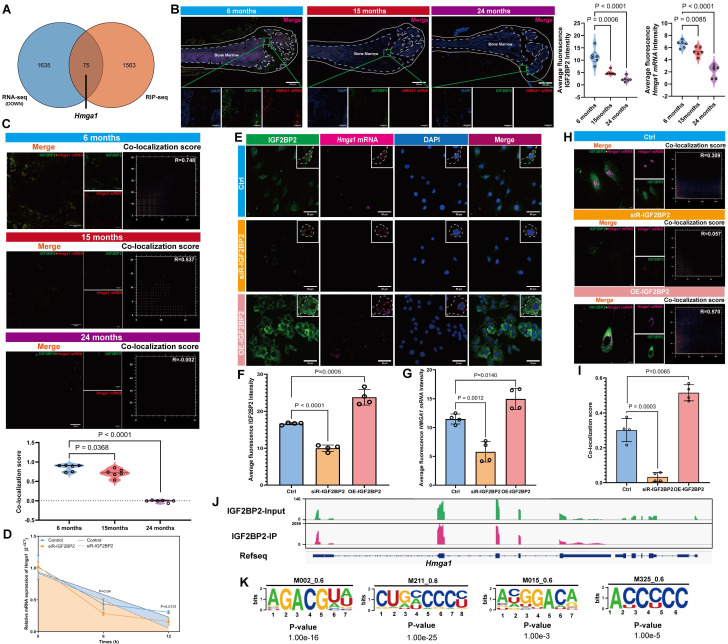
** IGF2BP2 regulates *Hmga1* mRNA stability in MSCs.** (A) Venn diagram of RNA-seq and RIP-seq overlaps. (B) Confocal imaging of IGF2BP2 (green), *Hmga1* mRNA (red), and nuclei (blue) in bone tissue (scale bars: 500 μm main, 100 μm insets). (C) IGF2BP2-*Hmga1* mRNA co-localization analysis. (D) *Hmga1* mRNA decay kinetics following actinomycin D treatment (control: blue; IGF2BP2-KD: yellow). (E-G) *In vitro* IGF2BP2-*Hmga1* mRNA co-localization (scale bars: 500 μm main, 100 μm insets). White/orange dashed lines demarcate cellular/nuclear boundaries. (H, I) Co-localization quantitation. (J) IGV browser tracks of IGF2BP2 binding to *Hmga1* mRNA. (K) IGF2BP2-binding motif at *Hmga1* mRNA. The results are shown as means ± SD, n ≥ 3 independent experiments; two-tailed Student's *t*-test.

Integrated analysis of RNA-seq and RIP-seq data identified *Hmga1* as a top candidate target of IGF2BP2 (Figure 4A, Figure S8B, Table S5). *Hmga1* expression was notably elevated in MSCs compared to other bone tissue cell types (Figure S8A).

An age-related decline in HMGA1 expression was observed via IHC and RT-qPCR (Figure S9A, B), accompanied by reduced fluorescence intensity of both IGF2BP2 and *Hmga1* mRNA in bone tissue from aged mice (Figure 4B). Critically, co-localization analysis revealed attenuated IGF2BP2-*Hmga1* mRNA interactions in older animals (Figure 4C). RIP-qPCR on mouse bone tissue confirmed significantly weakened binding of IGF2BP2 to *Hmga1* mRNA in aged mice (Figure S9C).

*In vitro*, *Hmga1* mRNA levels correlated positively with IGF2BP2 expression (Figure S10A, B). Actinomycin D chase assays demonstrated that* Hmga1* mRNA decayed more rapidly upon *Igf2bp2* knockdown, indicating reduced mRNA stability (Figure 4D). Modulation of *Igf2bp2* expression altered both the fluorescence intensity and subcellular localization of *Hmga1* mRNA: knockdown estrained both IGF2BP2 and *Hmga1* mRNA, decreased their co-localization, and reduced overall fluorescence signal; overexpression increased cytoplasmic accumulation of both IGF2BP2 and *Hmga1* mRNA, decreased in nuclear accumulation, enhanced their co-localization, and elevated overall fluorescence signals (Figure 4E-I). IGV visualization confirmed multiple binding sites between IGF2BP2 and *Hmga1* mRNA (Figure 4J), from which consensus binding motifs were derived (Figure 4K). In senescence models, IGF2BP2 knockdown reduced HMGA1 protein levels, while its overexpression did not significantly alter HMGA1 expression (Figure S10C, D).

### An IGF2BP2 point mutation promotes MSC senescence via HMGA1 regulation

**Figure 5:**
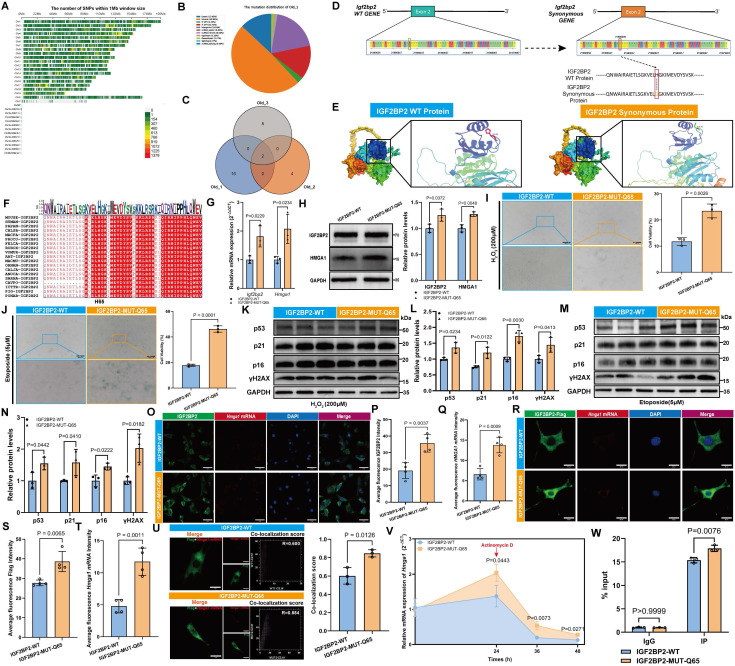
** IGF2BP2 mutation exacerbates senescence via HMGA1 dysregulation.** (A) Genome-wide SNP density across chromosomes (1 Mb bins). (B) Genomic distribution of SNPs. (C) Venn diagram of Old group sample overlaps. (D) WT versus MUT *Igf2bp2* sequences (green/red boxes: site 21980040). (E) Predicted 3D structures of WT/MUT IGF2BP2. (F) Cross-species IGF2BP2 sequence conservation (red: conserved residues; orange box: position 65). (G)* Igf2bp2* and *Hmga1* mRNA levels in WT/MUT cells. (H) Western blot and densitometry of IGF2BP2/HMGA1 (normalized to GAPDH). (I, J) SA-β-Gal staining (scale bars: 2000 μm main). (K-N) Western blot analysis of senescence markers. (O-Q) IGF2BP2-*Hmga1* mRNA co-localization in MUT MSCs (scale bar: 50 μm). (R-T) Flag-*Hmga1* mRNA co-localization (scale bar: 20 μm). (U) Co-localization quantitation. (V) *Hmga1* mRNA decay kinetics in WT/MUT cells post actinomycin D (red arrow: treatment onset). (W) RIP-qPCR validation of IGF2BP2-*Hmga1* mRNA binding. The results are shown as means ± SD, n ≥ 3 independent experiments; two-tailed Student's *t*-test.

We conducted a comprehensive analysis of single nucleotide polymorphisms (SNPs) across the entire genome of mouse BM-MSCs in Old Group. To investigate their distribution, we mapped the entire H. virgare genome using a sliding window size of 1 Mb (megabase pair) (Figure 5A). The results revealed non-uniform distribution across chromosomes, with higher densities on Chr7, Chr8, and Chr17. Most SNPs were located in intronic regions, followed by intergenic and 3'UTRs; only 13.02% resided in exons (Figure 5B). Analysis of the *Igf2bp2* gene identified two recurrent SNPs in aged samples: an intronic variant (Chr16:21,946,491 T>G) and an exonic non-synonymous mutation (Chr16:21,980,040 T>G) (Figure 5C). The latter results in a change from histidine (HIS) to glutamine (GLN) at amino acid position 65 (H65Q) in the IGF2BP2 protein (Figure 5D). In contrast, young samples carried a synonymous mutation at the same locus (Chr16:21,980,040 T>C, H65H) (Table S6). Structural modeling illustrated conformational differences between wild-type (WT) and mutant (MUT) proteins (Figure 5E). The interspecies sequence conservation analysis revealed that amino acid 65 is highly conserved across multiple species (Figure 5F). Cross-referencing with human databases revealed that the mouse Chr16:21980040 (T>G) mutation corresponds to human Chr3:185823197 (T>A) (rs1039767729). Population-level mutation frequencies were obtained across different age groups from gnomAD database. The results indicate that the mutation frequency is 0.04% in individuals aged ≤ 45 years and 0.05% in those over 45 years. Notably, the mutation frequency in males is 0.002%, whereas it is only 0.001% in females, which shows age- and sex-dependent variation in allele frequency (Table S7).

Introduction of the H65Q mutation (MUT) in IGF2BP2 increased its RNA and protein abundance, as well as HMGA1 expression, compared to WT (Figure 5G, H). Functionally, MUT-expressing MSCs exhibited enhanced senescence across models, with elevated SA-β-gal-positive rates (Figure 5I, J) and increased senescence marker expression (Figure 5K-N). These findings suggested that mutations in IGF2BP2 compromise the ability of wild-type IGF2BP2 to inhibit cellular senescence. IF analysis revealed that the intensity of IGF2BP2 and* Hmga1* mRNA in the MUT group was significantly higher compared to the WT group (Figure 5O-Q). To further evaluate the expression levels of exogenous protein and *Hmga1* mRNA, we quantified the intensity of the Flag-tagged protein. The results revealed stronger IGF2BP2 and *Hmga1* mRNA signals in MUT cells, with enhanced co-localization (Figure 5R-U). Actinomycin D assays showed accelerated decay of* Hmga1* mRNA in MUT-transfected cells (Figure 5V), while RIP-qPCR confirmed stronger binding of MUT IGF2BP2 to *Hmga1* mRNA (Figure 5W). These results suggest that the H65Q mutation enhances IGF2BP2's binding affinity to *Hmga1* mRNA rather than delaying its degradation.

### HMGA1 modulated DNA repair and influenced the aging of MSCs through its interaction with p53

**Figure 6:**
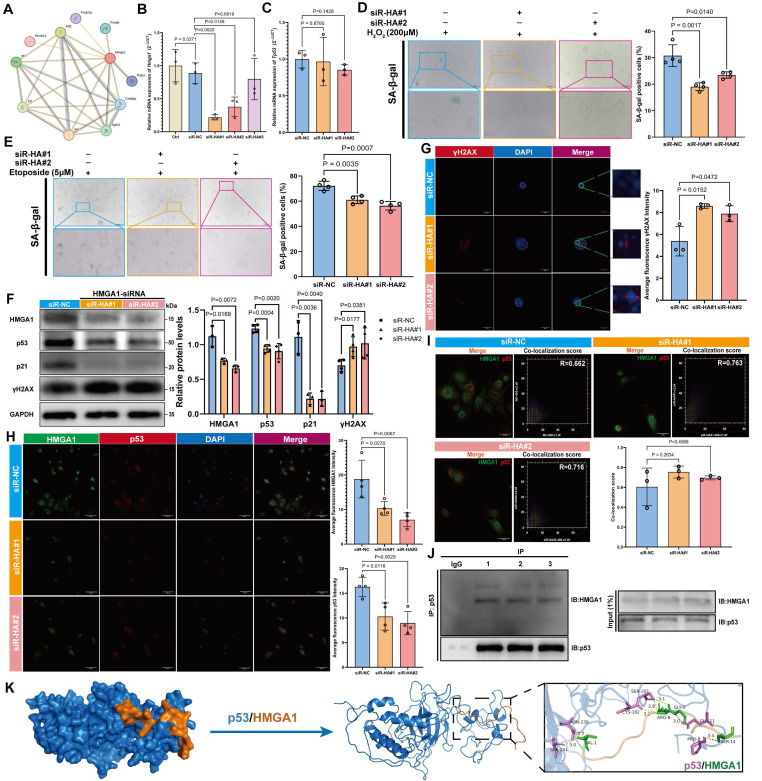
** HMGA1 attenuates senescence by targeting p53 in MSCs.** (A) HMGA1 PPI network. (B, C) *Hmga1* and *Tp53* mRNA levels post siRNA knockdown. (D, E) SA-β-Gal staining (scale bars: 2000 μm main). (F) Western blot and densitometry of HMGA1, p53, p21, and γH2AX (normalized to GAPDH). (G) HMGA1 (green)-p53 (red) co-localization (scale bar: 200 μm). (H) γH2AX foci analysis (scale bar: 50 μm). (I) Co-localization quantitation. (J) Co-IP validation of HMGA1-p53 interaction (triplicate experiments). (K) Molecular docking model of HMGA1 (orange)-p53 (blue) interaction; right panel highlights binding interfaces (violet: p53; green: HMGA1). The results are shown as means ± SD, n ≥ 3 independent experiments; two-tailed Student's *t*-test.

Protein-protein interaction (PPI) network analysis identified ten potential binding partners of HMGA1, among which TP53 (p53) was recognized as a key aging-related marker (Figure 6A). Three distinct siRNAs targeting HMGA1 were designed, and based on knockdown efficiency,* siR-#1* and *siR-#2* were selected for further experiments (Figure 6B). Knockdown of HMGA1 did not alter p53 expression at the mRNA level, suggesting that HMGA1 does not transcriptionally regulate p53 (Figure 6C). In both H_2_O_2_- and etoposide-induced senescence models, HMGA1 knockdown significantly reduced the proportion of SA-β-gal-positive cells (Figure 6D, E), indicating attenuation of cellular senescence. In normal MSCs, HMGA1 silencing led to decreased protein levels of p53 and p21, but a marked increase in γH2AX (Figure 6F), a finding corroborated by IF staining (Figure 6G, H). The co-localization between HMGA1 and p53 remained unchanged following HMGA1 knockdown (Figure 6I). Co-immunoprecipitation (Co-IP) using a p53-specific antibody confirmed a direct physical interaction between HMGA1 and p53 (Figure 6J). Molecular docking further predicted key interaction residues, including hydrogen bonds mediating HMGA1-p53 binding (Figure 6K).

### CWI1-2 attenuates cellular senescence and partially restores osteogenic differentiation *in vitro*

**Figure 7:**
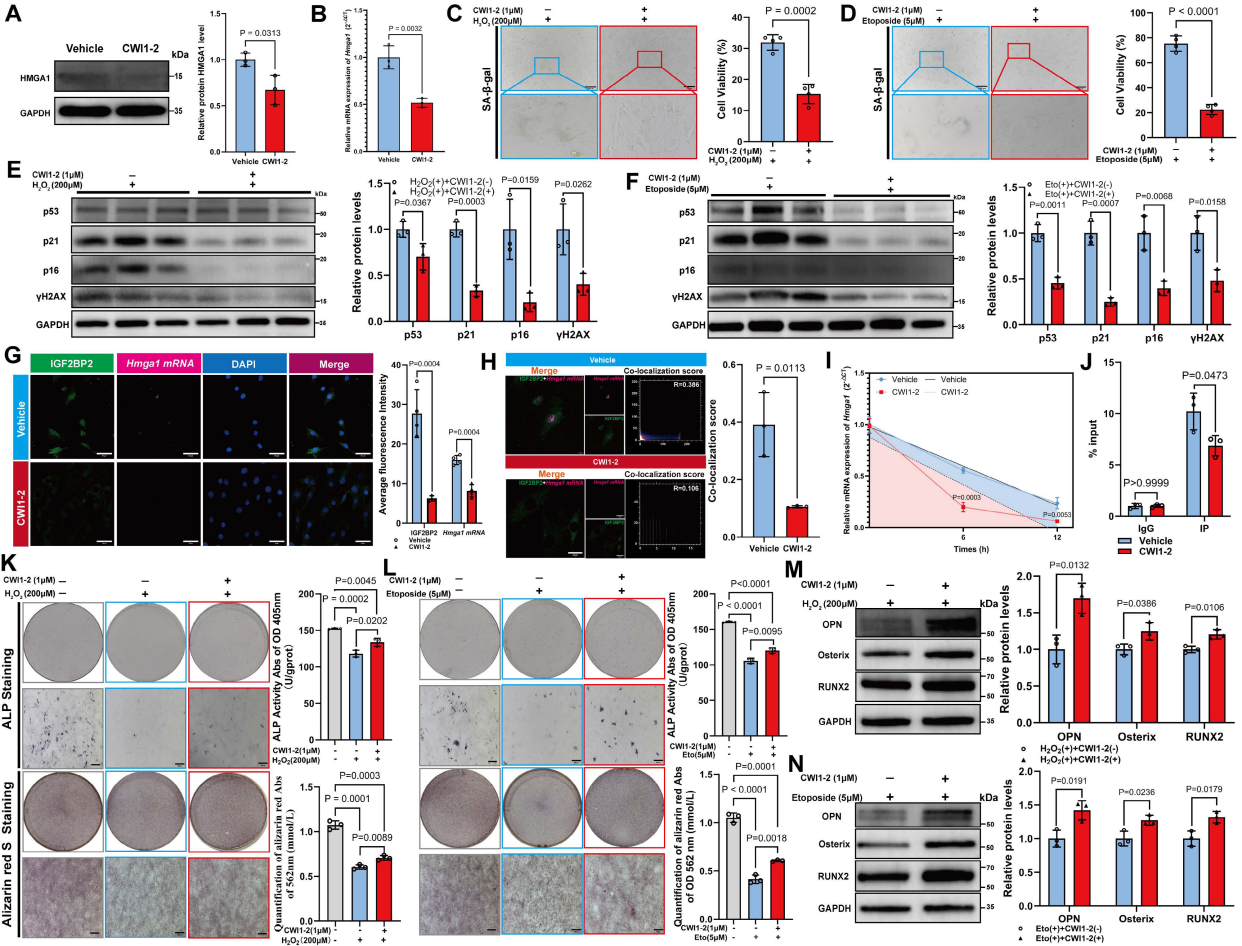
** CWI1-2 rejuvenates senescent MSCs and enhances osteogenesis.** (A) Western blot and densitometry of HMGA1 post CWI1-2 treatment (normalized to GAPDH). (B) *Hmga1* mRNA levels following CWI1-2 administration. (C, D) SA-β-Gal staining (scale bars: 2000 μm). (E, F) Western blot analysis of senescence markers. (G) IGF2BP2-*Hmga1* mRNA co-localization post treatment (scale bar: 50 μm). (H) Co-localization quantitation. (I) *Hmga1* mRNA stability assay with actinomycin D (Vehicle: blue; CWI1-2: red). (J) RIP-qPCR of IGF2BP2-*Hmga1* mRNA binding. (K, L) ALP/ARS staining and quantitation (scale bars: 2000 μm). (M, N) Western blot analysis of osteogenic markers. The results are shown as means ± SD, n ≥ 3 independent experiments; two-tailed Student's *t*-test.

Treatment with 1 μM CWI1-2 significantly reduced both mRNA and protein levels of HMGA1 (Figure 7A, B) and alleviated senescence in MSCs, as evidenced by reduced SA-β-gal positivity and senescence marker expression (Figure 7C-F). Furthermore, CWI1-2 decreased the fluorescence intensity of IGF2BP2 and *Hmga1* mRNA and reduced their co-localization (Figure 7G, H). Actinomycin D chase assays showed accelerated decay of *Hmga1* mRNA in CWI1-2-treated cells (Figure 7I), and RIP-qPCR confirmed diminished binding of IGF2BP2 to *Hmga1* mRNA (Figure 7J).

To evaluate the effect of CWI1-2 on osteogenic differentiation, senescent MSCs were subjected to osteogenic induction in the presence of CWI1-2. The compound enhanced ALP activity, mineralization, and osteogenic marker expression in both H₂O₂- and etoposide-induced models (Figure 7K-N), though the restorative effect remained partial compared to the control group.

### CWI1-2 ameliorates bone tissue degeneration and promotes bone regeneration in aged mice

**Figure 8:**
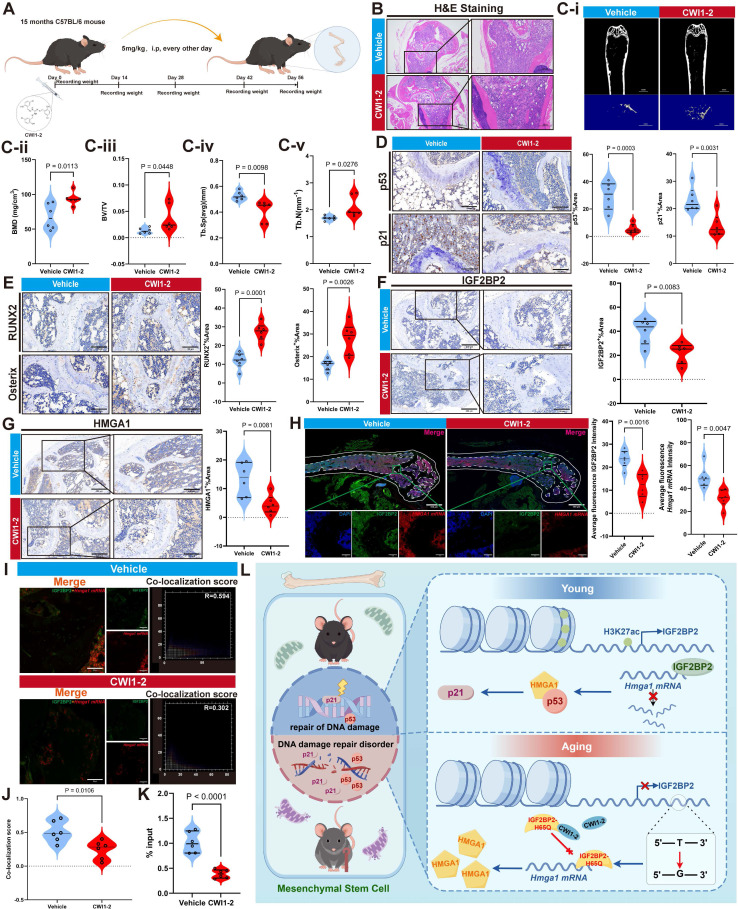
** CWI1-2 ameliorates bone aging in murine models.** (A) Schematic of CWI1-2 treatment in aging mice by Figdraw. (B) H&E-stained bone sections (scale bars: 500 μm main, 200 μm insets). (Ci-v) Micro-CT analysis of bone parameters. (D) IHC detection of p53/p21 (scale bar: 100 μm). (E) IHC of osteogenic markers RUNX2 and Osterix (scale bar: 100 μm). (F, G) IHC of IGF2BP2 and HMGA1 (scale bars: 200 μm main, 100 μm insets). (H) Confocal imaging of IGF2BP2 (green), *Hmga1* mRNA (red), and nuclei (blue) in bone (scale bars: 500 μm main, 50 μm insets). (I, J) Co-localization quantitation. (K) RIP-qPCR of IGF2BP2-*Hmga1* mRNA binding in bone tissues. (L) The model diagram of this study by Figdraw. The results are shown as means ± SD, n ≥ 3 independent experiments; two-tailed Student's *t*-test.

Body weight of mice was monitored throughout CWI1-2 treatment and showed no significant change (Figure 8A, Figure S11). CWI1-2-treated mice exhibited improved bone microarchitecture, with increased trabecular bone volume and repair of bone microstructure (Figure 8B, Ci-v), accompanied by reduced senescence markers (p53, p21 and p16) and elevated osteogenic markers (RUNX2, Osterix and p16) (Figure 8D, E, Figure S12A, B). These results indicate that CWI1-2 not only alleviates bone tissue aging but also enhances bone mass and regenerative capacity.

Both protein and mRNA levels of IGF2BP2 and HMGA1 were significantly lower in the CWI1-2 group compared to controls (Figure 8F-H, Figure S12C). Immunofluorescence analysis revealed reduced co-localization of IGF2BP2 with *Hmga1* mRNA (Figure 8I, J), and RIP-qPCR on bone tissue confirmed decreased binding of IGF2BP2 to *Hmga1* mRNA following CWI1-2 treatment (Figure 8K).

## Discussion

In this study, we profiled H3K27ac modifications in aged bone marrow-derived mesenchymal stem cells (BM-MSCs) and identified IGF2BP2 as a key downstream effector. We observed a pronounced age-related decline in H3K27ac levels in both bone tissue and cellular models of senescence, accompanied by reduced IGF2BP2 expression. Intriguingly, although both knockdown and overexpression of *Igf2bp2* alleviated MSC senescence *in vitro*, altered IGF2BP2 expression itself was not the primary driver of senescence. Further analysis revealed an age- and sex-associated point mutation in *Igf2bp2*, which exacerbates MSC aging by inducing functional alterations in the IGF2BP2 protein. Moreover, treatment with CWI1-2, a covalent inhibitor of IGF2BP2, attenuated MSC senescence *in vitro* and enhanced osteogenic differentiation and bone regeneration *in vivo* and *in vitro* (Figure 8L).

Age-related bone loss is closely linked to cellular senescence. Previous studies demonstrate that depleting senescent cells, dampening inflammation, and remodeling the senescent microenvironment can substantially improve bone mass and delay skeletal aging 31, 32. While earlier research emphasized osteoclast-mediated bone loss 33, this study highlights the crucial role of MSC senescence in age-related skeletal decline. This is consistent with recent findings by Dominik *et al.* 34, who identified senescent MSC populations in aged bone using the "SenMayo" gene signature. We also observed elevated DNA damage response (DDR) and DNA repair pathway activity in aged MSCs, indicative of functional decline.

Impaired DNA repair may exacerbate bone metabolic imbalance, tissue dysfunction, and cellular senescence. Genetic instability, a key driver of aging, leads to significant biological consequences. Accumulated DNA damage activates multiple signaling cascades and initiates the DDR, which detects structural DNA alterations and activates cell cycle checkpoints to allow time for repair. Unrepaired DNA damage may result in cellular senescence or apoptosis 35. Within the DDR, p53 plays a central role by recognizing specific promoter regions and promoting the expression of cell cycle inhibitors such as CDKN1A and pro-apoptotic factors. p53 is also involved in various DNA repair mechanisms 36. Although the DDR provides repair potential, growing evidence suggests that DNA damage engages the cGAS-STING axis and ATM-mediated NF-κB activation, leading to robust expression of inflammatory factors 37, which may accelerate aging. Beyond the p53/p21 pathway examined here, other signaling pathways—including the Hippo 38, AMPK, NF-κB, and SIRT1 pathways—also play crucial roles in regulating cellular functions, DDR, and aging 39.

In bone aging, the dynamic distribution of bone-resident cells is essential for regulating bone metabolism, particularly through interactions among osteoclasts, adipocytes, osteoblasts, and MSCs. These interactions alter the bone marrow microenvironment and promote shifts in differentiation pathways, ultimately leading to diminished bone regenerative capacity 40. Furthermore, aging bone tissue exhibits lipid droplet accumulation. Studies indicate that bone marrow adipocytes inhibit osteogenic differentiation of stem cells 41 and promote senescence in osteoblasts and osteoclasts via the AKT/mTOR pathway 42. Additionally, extracellular matrix-related pathways are critical for MSC regenerative capacity and contribute significantly to tissue repair 43.

Epigenetic dysregulation, especially changes in histone modifications, is increasingly recognized as a driver of aging 44. In hematopoietic stem cells (HSCs), H3K27ac is associated with cell activation, apoptosis, and histone modification control, with aged HSCs showing altered H3K27ac patterns at promoters and enhancers 45. Our work extends this concept to BM-MSCs, demonstrating that H3K27ac may have a more pronounced regulatory effect on BM-MSCs than on other cell types within bone tissue. Moreover, the loss of histone H3K27ac disrupts enhancer-driven transcription, with IGF2BP2 serving as a key downstream mediator. The conserved role of IGF2BP2 in RNA stabilization 46 and its age-related decline suggest a unified mechanism connecting epigenetic changes to post-transcriptional dysregulation in aging.

Our previous work identified IGF2BP2 dysregulation in osteoporosis 47. *In vitro*, *Igf2bp2* knockdown promoted osteogenic differentiation 14, consistent with our observations in senescent MSCs. IGF2BP2 also regulates cell proliferation. IGF2BP2 also regulates cell proliferation 48; thus, exogenous IGF2BP2 may help restore the proliferative capacity of senescent MSCs, expanding the progenitor pool available for osteogenic commitment. This may explain the apparent discrepancy wherein IGF2BP2 overexpression enhanced osteogenic capacity despite downregulating some osteogenic markers.

The IGF2BP2-HMGA1-p53 axis represents a novel regulatory node in MSC senescence. IGF2BP2 stabilizes *Hmga1* mRNA, linking post-transcriptional control to aging. Although IGF2BP2's role in aging is documented 49-51, its mechanisms remain incompletely understood. Here, its interaction with HMGA1 reveals an additional layer of aging control. HMGA1, which modulates chromatin architecture, is known to influence aging 52, 53. We found that HMGA1 knockdown increased γH2AX levels in normal MSCs, supporting a role in DNA damage repair 54. Mechanistically, HMGA1 interacts with the C-terminal domain of p53 family proteins to modulate downstream signaling 55, 56. HMGA1 knockdown reduced p53 and p21 levels without affecting HMGA1-p53 co-localization, suggesting that HMGA1 may facilitate p53 protein degradation.

Notably, both knockdown and overexpression of IGF2BP2 ameliorated senescence, indicating that functional activity—not merely expression level—determines its role. Suo *et al.* 57 reported reduced IGF2BP2 in senescence and showed that its deletion more strongly affects young mice, implying an age-related loss of function. Our results suggest that IGF2BP2 dysregulation is a consequence rather than a cause of aging, with endogenous IGF2BP2 becoming progressively impaired over time—a mechanism distinct from conventional inhibition/activation models 58, prompting further investigation into the role of IGF2BP2 in the aging process.

Accumulated mutations contribute to genetic disorders and cancers 59 and vary tissue-specifically with age, influencing disease incidence 60. For example, numerous LMNA mutations are associated with degenerative diseases like Hutchinson-Gilford progeria syndrome 61. Interestingly, Suo *et al.* 57 reported that although the expression of IGF2BP2 decreases with age, deletion of the gene in aged mice enhances hematopoietic stem cell activity, suggesting that the functional role of IGF2BP2 transitions from beneficial in youth to detrimental in old age. Research has suggested that this functional shift may be associated with mutations arising from DDR processes 62, 63. Based on these findings, we propose that age-associated mutations in IGF2BP2 may contribute to functional decline and cellular dysfunction. Therefore, identifying the specific mutation sites that accumulate during aging is of critical importance.

Here, we identified a critical point mutation in *Igf2bp2*-H65Q-present in both mouse and human samples, with frequencies varying by age and sex: higher in middle-aged and elderly individuals than in adolescents, and more common in males. This suggests a link between MSC senescence and osteoporosis in older men. Mechanistically, the H65Q mutation enhances IGF2BP2 binding to *Hmga1* mRNA, increasing HMGA1 expression and accelerating MSC senescence via p53 activation. Hence, we term this mutant a “bad protein.” Although we identified this mutation, larger clinical samples are needed to clarify the relationship between *Igf2bp2* mutation rates and bone aging in patients. Furthermore, due to the relatively low prevalence of this mutation in the human population, its physiological impact on bone aging appears to be less pronounced and less significant in humans compared to that observed in murine models, thereby necessitating further epidemiological studies for validation.

We also noted that IGF2BP2 localizes to both the cytoplasm and nucleus in MSCs. Overexpression of *Igf2bp2* reduced nuclear IGF2BP2 levels. In senescent cells, IGF2BP2 overexpression did not alter HMGA1 levels. We hypothesize that ectopic cytoplasmic overexpression of* Igf2bp2* elevated its mRNA levels, thereby suppressing endogenous nuclear *Igf2bp2* expression. Since *Hmga1* mRNA—regulated by IGF2BP2—is primarily nuclear, reduced nuclear IGF2BP2 may destabilize *Hmga1* mRNA, lowering nuclear HMGA1 levels after IGF2BP2 overexpression and potentially mitigating further senescence.

Notably, H3K27ac levels decreased during senescence, reducing IGF2BP2 expression. This appears contradictory to the anti-senescence effect of *Igf2bp2* knockdown. Based on prior research, we propose that changes in IGF2BP2 expression are not the direct cause of senescence. Instead, senescence induction (e.g., via DNA damage) may trigger IGF2BP2 mutations, generating a “bad” protein that promotes senescence through the HMGA1/p53/p21 pathway. H3K27ac reduction may be a compensatory response attenuating the mutation's effects.* Igf2bp2* knockdown reduces “bad” protein levels, alleviating senescence and improving osteogenesis. Overexpression may suppress the mutant protein, allowing a functional (“good”) IGF2BP2 to promote proliferation, mitigate senescence, and supply higher-quality osteoprogenitor cells.

Comparing IGF2BP2 overexpression and inhibition, we found inhibition conferred more stable anti-aging effects. Accordingly, we used CWI1-2, a covalent inhibitor targeting the IGF2BP2 KH3-4 domain 64—which disrupted mutant IGF2BP2/*Hmga1* mRNA binding, attenuated senescence, promoted osteogenesis, and reduced bone loss in aging models.

Several limitations remain: clinical correlations between H3K27ac/IGF2BP2/HMGA1 and human bone aging are lacking; the tissue specificity and physiological relevance of the low-frequency H65Q mutation require further study; and potential synergies between H3K27ac restoration and IGF2BP2 modulation remain unexplored. Our study relied solely on murine models, which may not fully recapitulate human bone aging. Meanwhile, our research has primarily focused on *in vitro* cell studies, and there remains a lack of in-depth *in vivo* experiments to further investigate the effects of IGF2BP2 on aging organisms and its underlying mechanisms. Additionally, the therapeutic efficacy of CWI1-2 in large-animal models remains untested. These issues also highlight valuable directions for future research, such as further investigating the *in vivo* effects of IGF2BP2 on bone aging and elucidating the underlying mechanisms through the development of IGF2BP2 knockout mouse models. Moreover, advanced methodologies, including bone organoids 65-67, can be employed to further validate the therapeutic potential and mechanism of CWI1-2, thereby opening new avenues for the translational exploration of IGF2BP2 and MSCs 68.

In conclusion, this study elucidates age-related declines in H3K27ac and identifies IGF2BP2 as a key epigenetic effector in bone aging. We further uncover a mutation-driven mechanism of IGF2BP2 dysfunction in senescence, providing new insights into therapeutic strategies for ameliorating age-related bone loss and enhancing regeneration.

## Supplementary Material

Supplementary figures and tables.

## Abbreviations

ALP: Alkaline phosphatase
BM-MSCs: Bone marrow-derived mesenchymal stem cells
BMD: Bone mineral density
BV/TV: Bone volume/tissue volume
ChIP-seq: Chromatin immunoprecipitation sequencing
Co-IP: Co-immunoprecipitation
DEGs: Differentially expressed genes
DMSO: Dimethyl sulfoxide
FBS: Fetal bovine serum
FISH: Fluorescence in situ hybridization
GO: Gene Ontology
H3K27ac: Histone H3 lysine 27 acetylation
HMGA1: High mobility group A1
IGF2BP2: Insulin-like growth factor 2 mRNA-binding protein 2
IHC: Immunohistochemistry
KEGG: Kyoto Encyclopedia of Genes and Genomes
MSCs: Mesenchymal stem cells
mRNA: Messenger RNA
Micro-CT: Micro-computed tomography
PBS: Phosphate-buffered saline
RIP-seq: RNA immunoprecipitation sequencing
RNA-seq: RNA sequencing
RT-qPCR: Real-time quantitative polymerase chain reaction
SA-β-gal: Senescence-associated β-galactosidase
SNP: Single nucleotide polymorphism
Tb.N: Trabecular number
Tb.Sp: Trabecular separation
