# Supplementary material for: H3K27 Acetylation-driven IGF2BP2 Mutates during the Aging of MSCs, thereby Influencing Osteogenic Differentiation and Bone Aging
Source: Int J Biol Sci. 2026 Jan 1;22(1):142–60. doi: 10.7150/ijbs.122708 (PMC12681744; doi:10.7150/ijbs.122708)
Supplement: Supplementary file 1 — Supplementary figures and tables. [file ijbsv22p0142s1.zip › Supplementary materials.pdf]

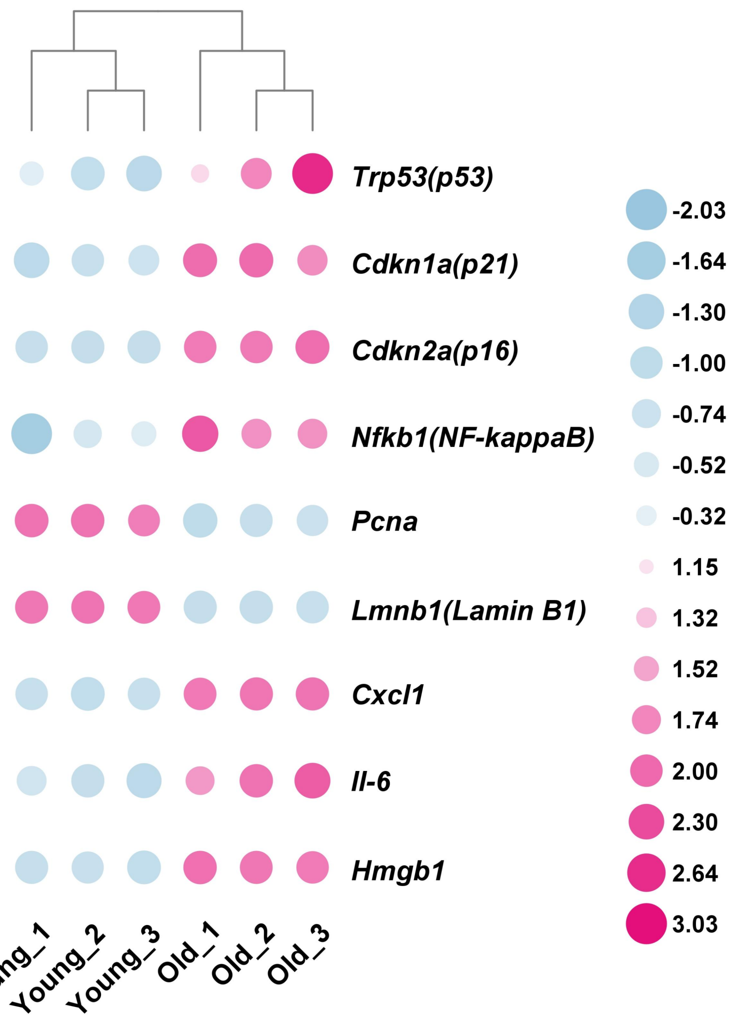

Supplemental Figure 1. Heatmap of Aging Markers in RNA-seq

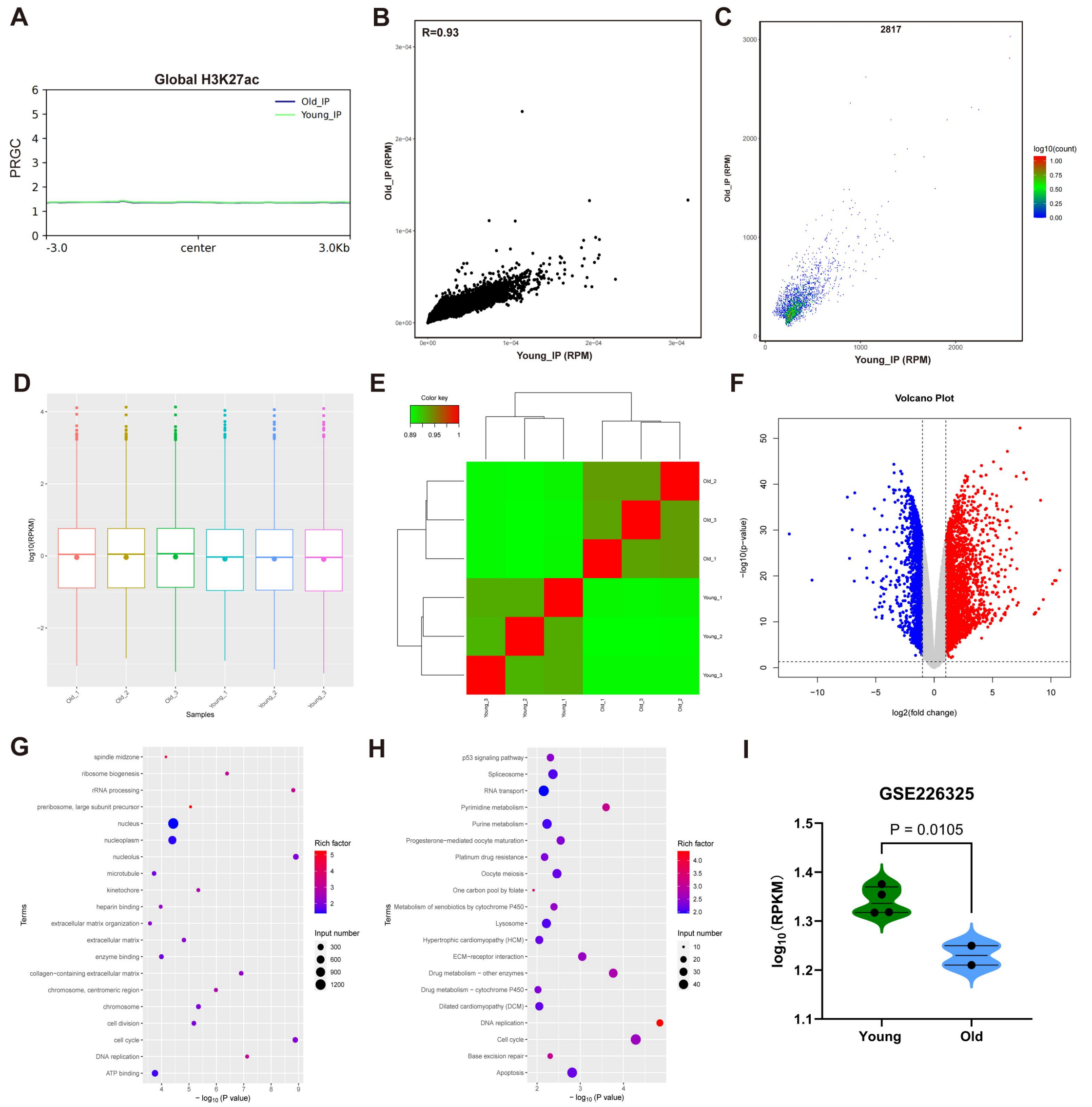

Supplemental Figure 2. Integrated Analysis of H3K27ac ChIP-seq and RNA-seq

(A) Aggregate plots showed the distribution of the average H3K27ac levels throughout the genome in Young Group and Old Group. (B)Scatter plot correlating H3K27ac occupancy levels between Young and Old groups. (C) Scatter plot comparing H3K27ac occupancy at enhancer regions across groups. (D) Box plot representation of RPKM values. (E) Hierarchical clustering of transcriptomes across samples, with a green-to-red gradient reflecting low-to-high inter-sample correlation coefficients. (F) Volcano Plots of Gene Expression Profiles. (G, H) GO and KEGG enrichment analyses of DEGs, visualized as bubble charts. (I) *Igf2bp2* expression levels in validation dataset GSE226325. The results are shown as means ± SD, n ≥ 3 independent experiments; two-tailed Student's *t*-test.

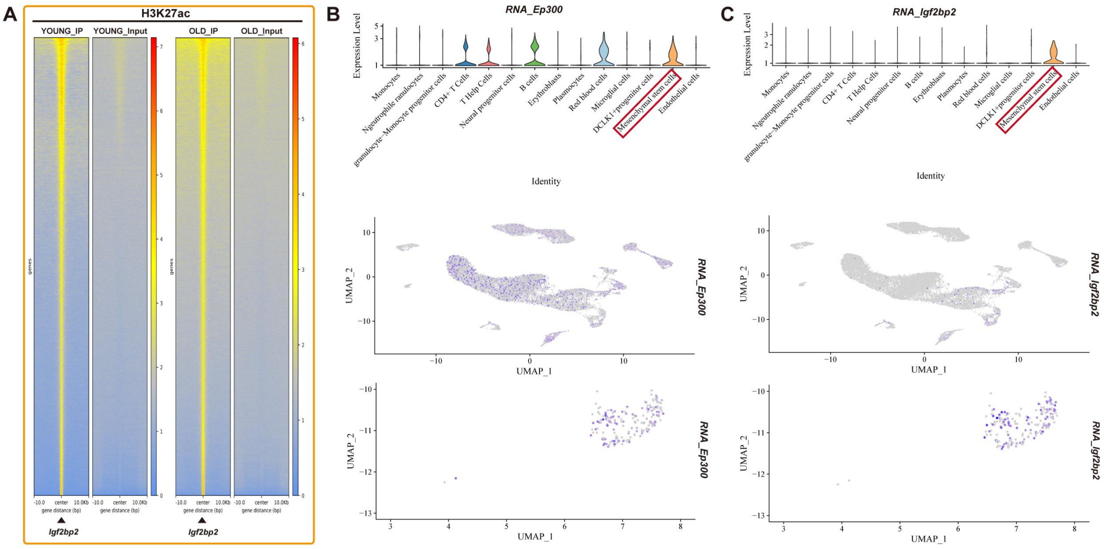

Supplemental Figure 3. Integrated Analysis of H3K27ac-draven *Igf2bp2* in ChIP-seq and Single-cell Analysis

(A) H3K27ac signal distribution relative to *Igf2bp2* midpoints. (B, C) Spatial expression patterns of *Ep300* and *Igf2bp2* mRNA across cell populations. Left: Violin plots (x-axis: cell types; y-axis: expression levels; red boxes highlight MSCs). Middle: UMAP projections of molecule expression. Right: MSC-specific expression gradients (gray-to-purple gradient indicates low-to-high expression). The results are shown as means ± SD, n ≥ 3 independent experiments; two-tailed Student's *t*-test.

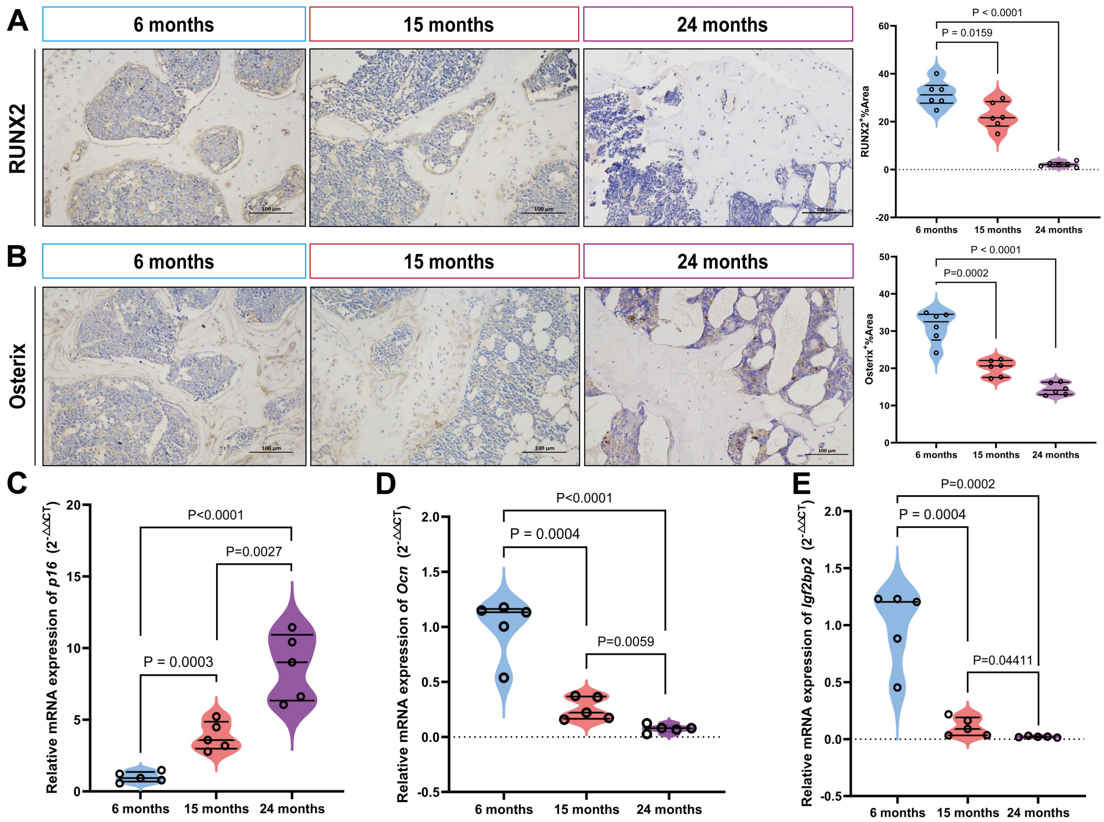

Supplemental Figure 4. Analysis of Osteogenic Differentiation Markers and p16 in Bone Tissue Samples from Mice of Varying Ages.

(A) IHC demonstrated the expression of RUNX2 in bone tissue samples (scale bars: 100 μm). (B) IHC demonstrated the expression of Osterix in bone tissue samples (scale bars: 100 μm). (C) *p16* mRNA levels in bone tissue samples. (D) *Ocn* mRNA levels in bone tissue samples. (E) *Igf2bp2* mRNA levels in bone tissue samples. The results are shown as means ± SD, n ≥ 5 independent experiments; two-tailed Student's *t*-test.

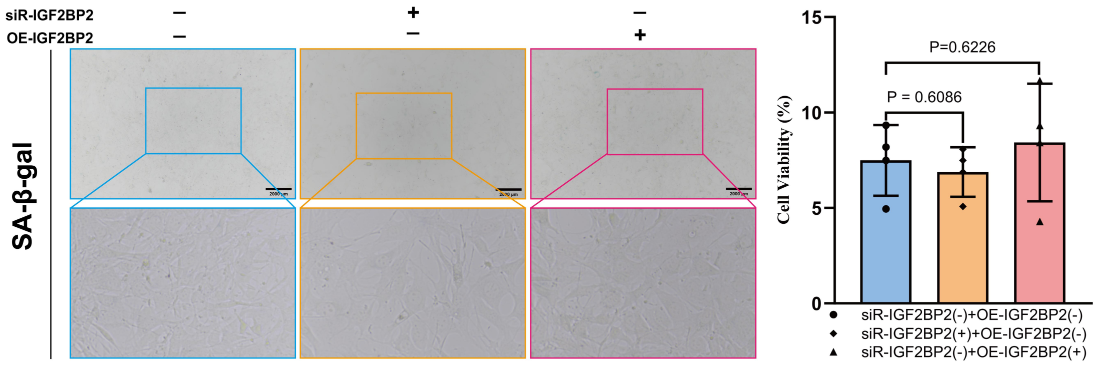

Supplemental Figure 5. SA-β-gal staining following The Knockdown or Overexpression of IGF2BP2 in Normal MSCs

Results of SA-β-gal staining (scale bars: 2000 μm).The results are shown as means ± SD, n ≥ 3 independent experiments; two-tailed Student's *t*-test.

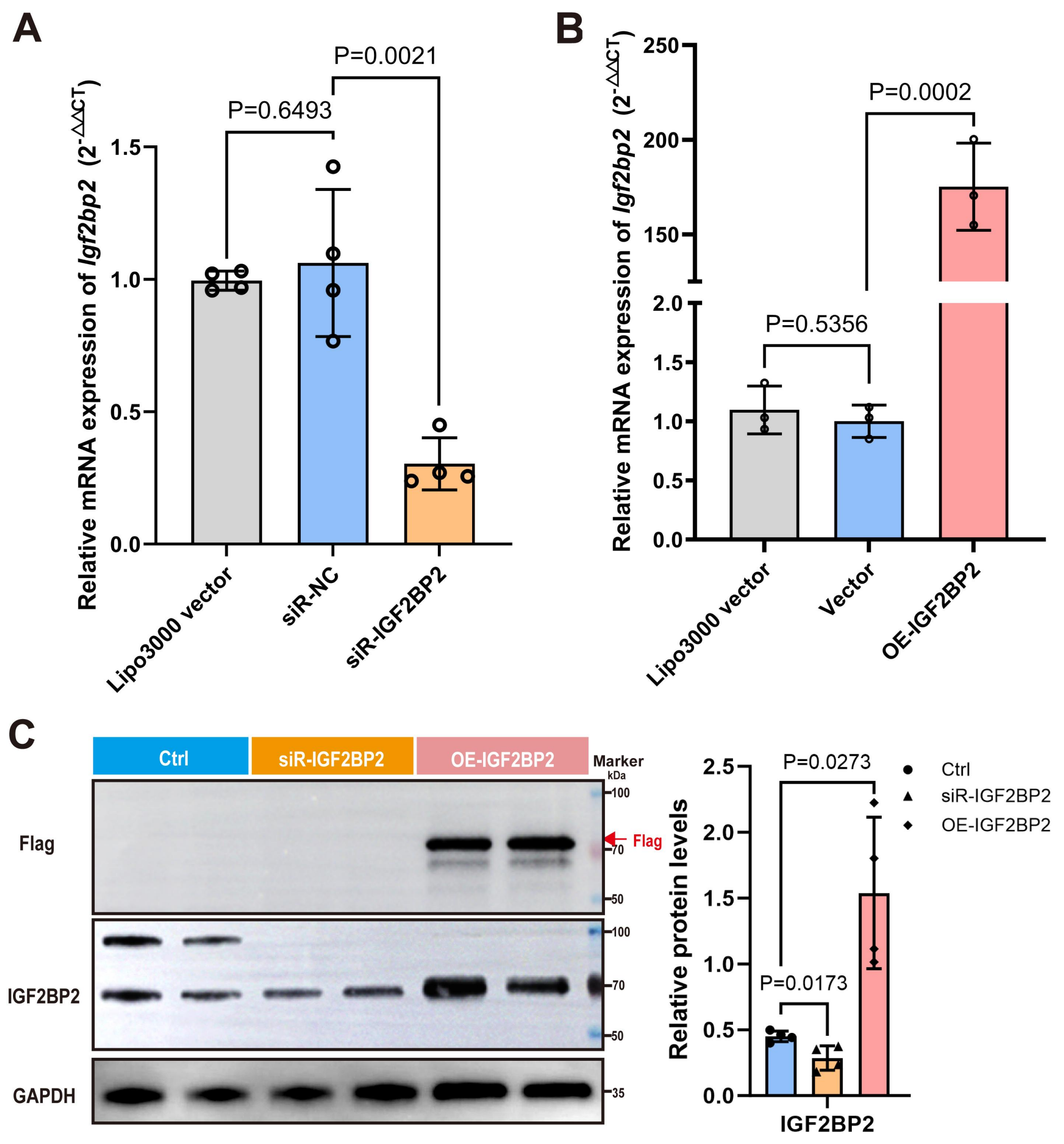

Supplemental Figure 6. The mRNA Level of *Igf2bp2* After Down-regulation or Overexpression *Igf2bp2* in Normal MSCs.

(A) *Igf2bp2* mRNA levels post IGF2BP2 knockdown. (B) *Igf2bp2* mRNA levels post IGF2BP2 overexpression. (C)Western blot and densitometry of Flag-tagged IGF2BP2, IGF2BP2 (normalized to GAPDH). The results are shown as means ± SD, n ≥ 3 independent experiments; two-tailed Student's *t*-test.

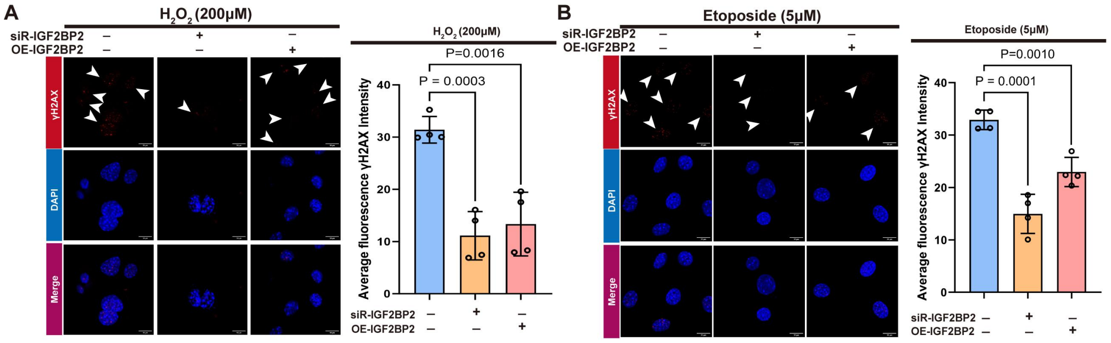

Supplemental Figure 7. γH2AX IF on Senescent MSC

(A, B) γH2AX IF analysis (scale bar: 50 μm). The results are shown as means ± SD, n ≥ 3 independent experiments; two-tailed Student's *t*-test.

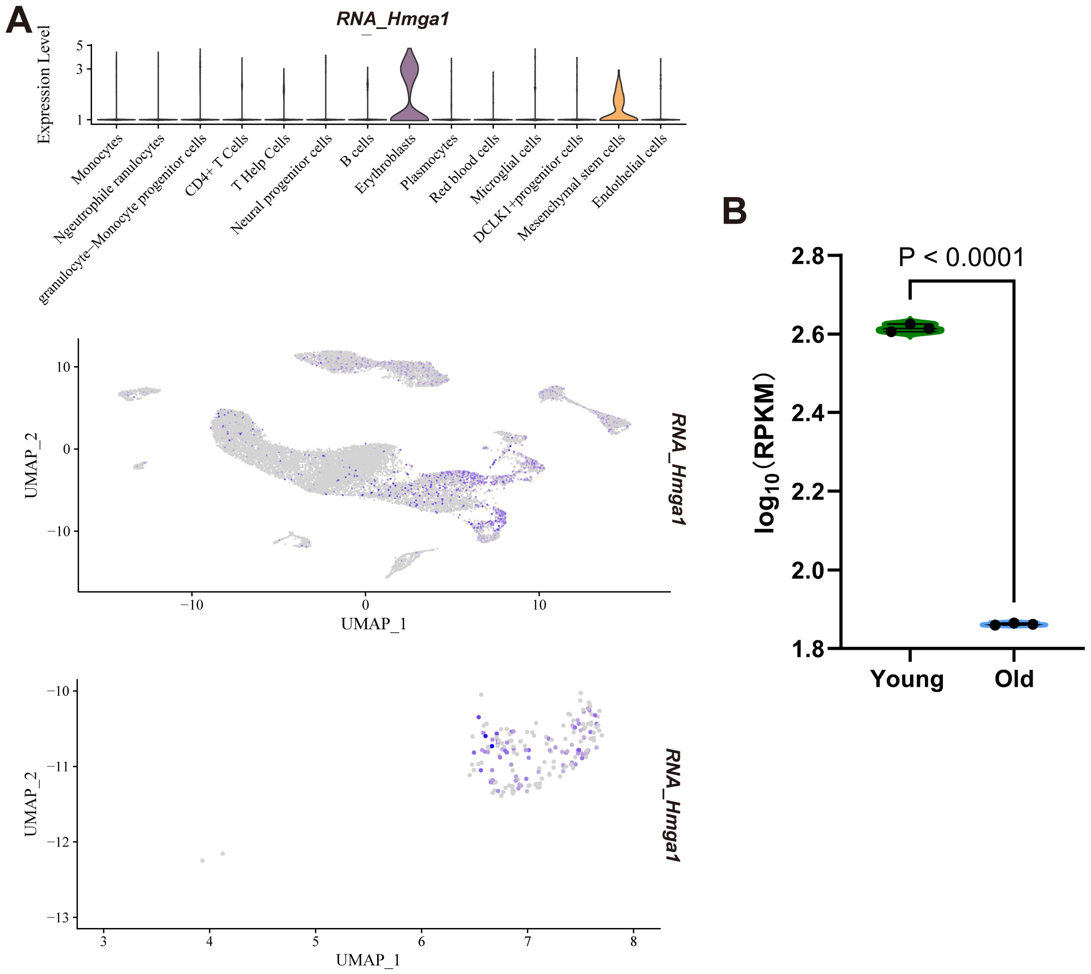

Supplemental Figure 8. Expression of *Hmga1* in Cell Population and RNA-seq Analysis

(A) *Hmga1* mRNA expression across cell populations. (B) Expression of *Hmga1* in RNA-seq analysis. The results are shown as means ± SD, n ≥ 3 independent experiments; two-tailed Student's *t*-test.

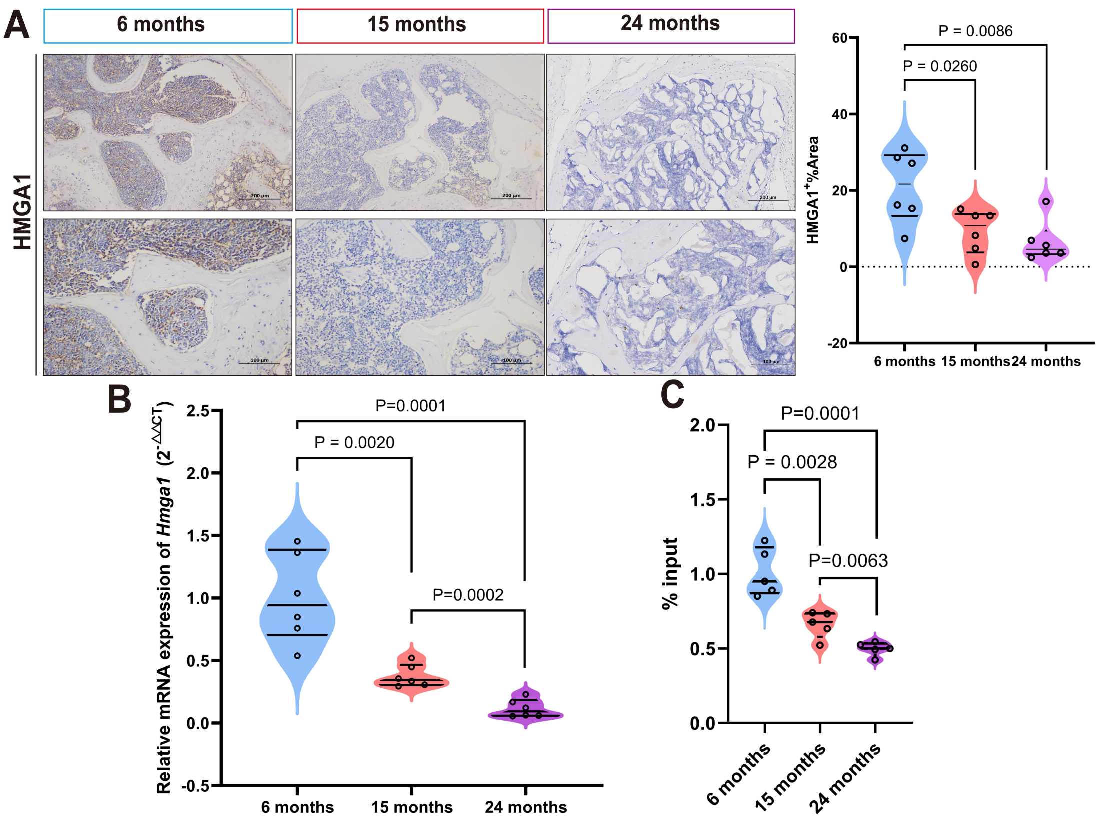

Supplemental Figure 9. Analysis of HMGA1 in Bone Tissue Samples from Mice of Varying Ages.

(A) IHC demonstrated the expression of HMGA1 in bone tissue samples (scale bars: 200 μm, enhanced scale bars: 100 μm). (B) *Hmga1* mRNA levels in different aging group. (C) RIP-qPCR validation of IGF2BP2-*Hmga1* mRNA binding in different aging group. The results are shown as means ± SD, n ≥ 5 independent experiments; two-tailed Student's *t*-test.

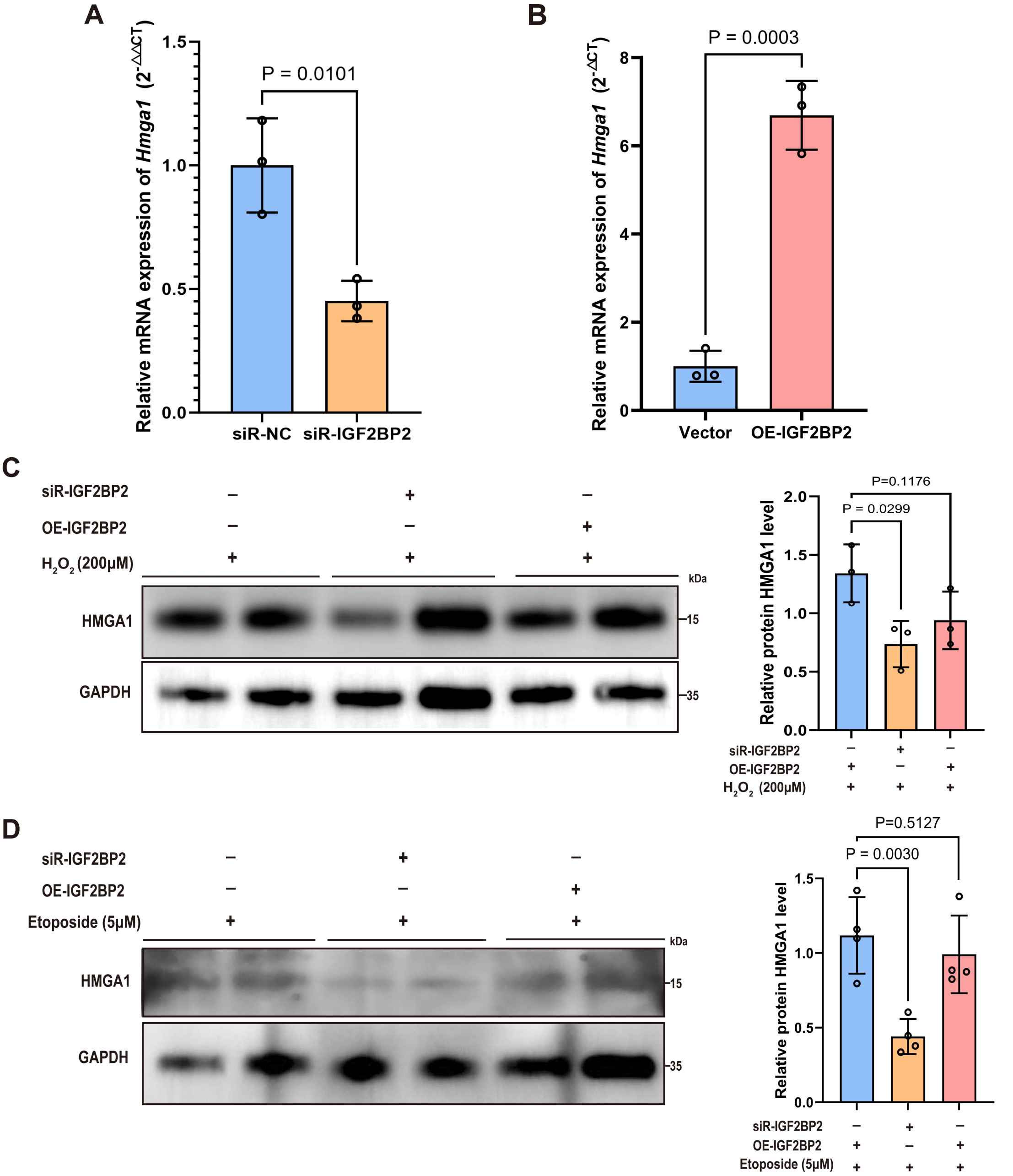

Supplemental Figure 10. The HMGA1 Levels following IGF2BP2 Knockdown and Overexpression in Normal MSCs and in vitro Cellular Senescence Models. (A) *Hmga1* mRNA levels post *Igf2bp2* knockdown. (B) *Hmga1* mRNA levels post *Igf2bp2* overexpression in normal MSCs. (C, D) Western blot and densitometry of HMGA1 (normalized to GAPDH) in vitro cellular senescence models. The results are shown as means ± SD, n ≥ 3 independent experiments; two-tailed Student's *t*-test.

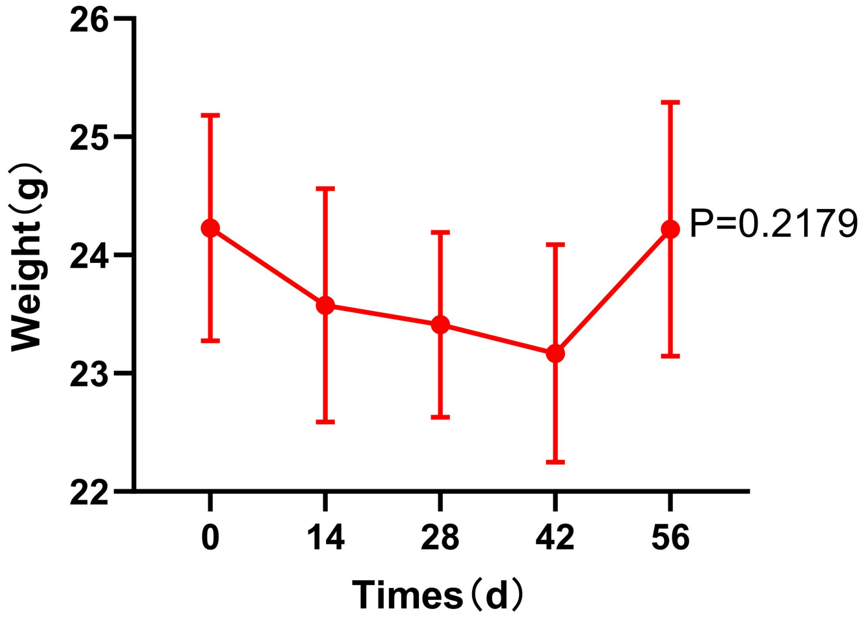

Supplemental Figure 11. Body Weight Measurements of Mice at Various Time Points following CWI1-2 Administration

The results are shown as means ± SD, n ≥ 6 independent experiments; One-way analysis of variance (ANOVA) was used to perform.

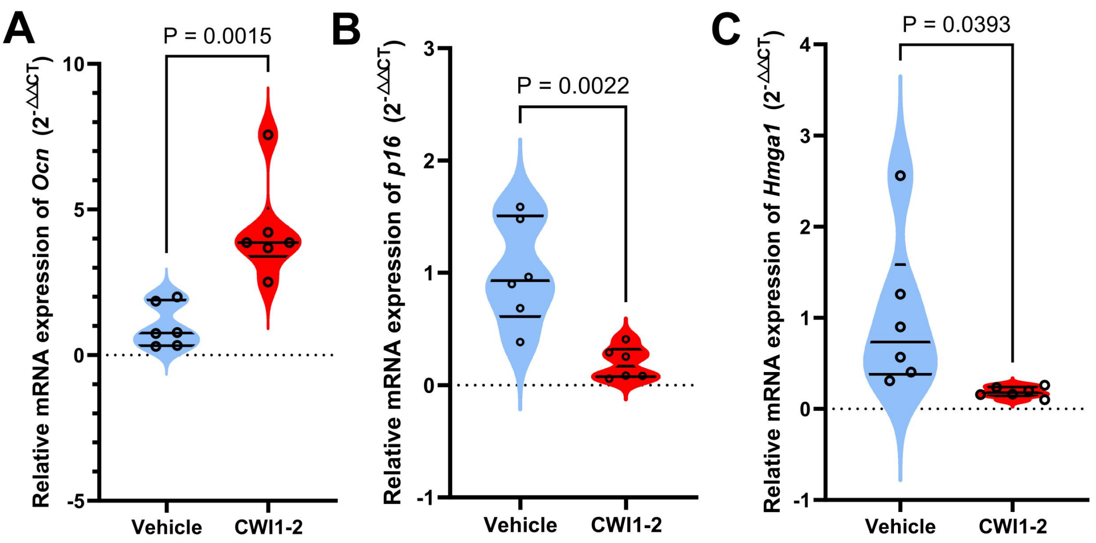

Supplemental Figure 12. *Ocn*, *p16* and *Hmga1* mRNA Levels of Bone Tissues following CWI1-2 Administration

(A) *Ocn* mRNA levels of bone tissues following CWI1-2 administration. (B) *p16* mRNA levels of bone tissues following CWI1-2 administration. (C)*Hmga1* mRNA levels of bone tissues following CWI1-2 administration. The results are shown as means ± SD, n ≥ 6 independent experiments; two-tailed Student's *t*-test.

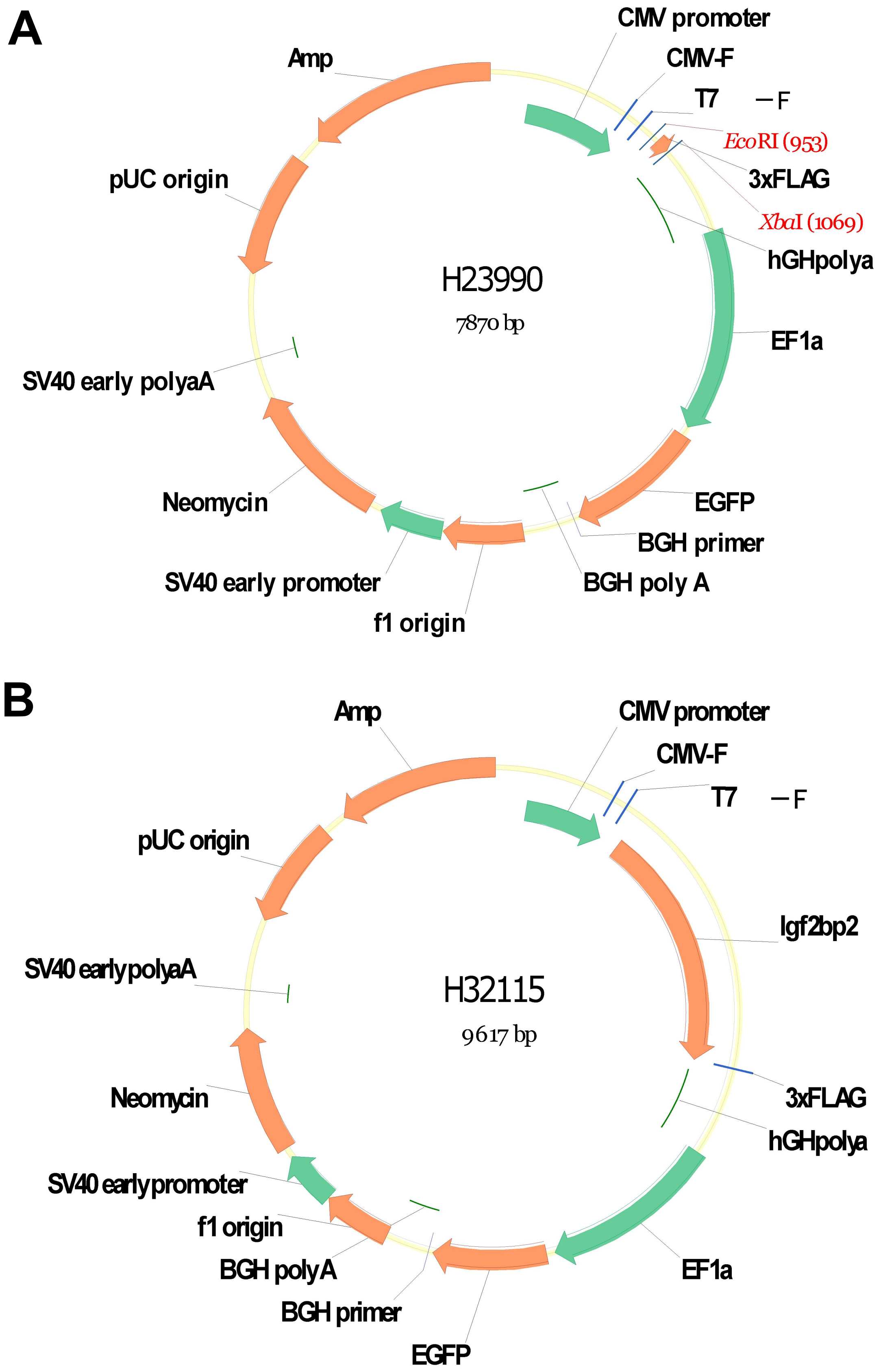

Supplemental Figure 13. *Igf2bp2* No-Load and Overexpression Plasmid Maps

(A) No-Load plasmid maps. (B) Overexpression plasmid maps.

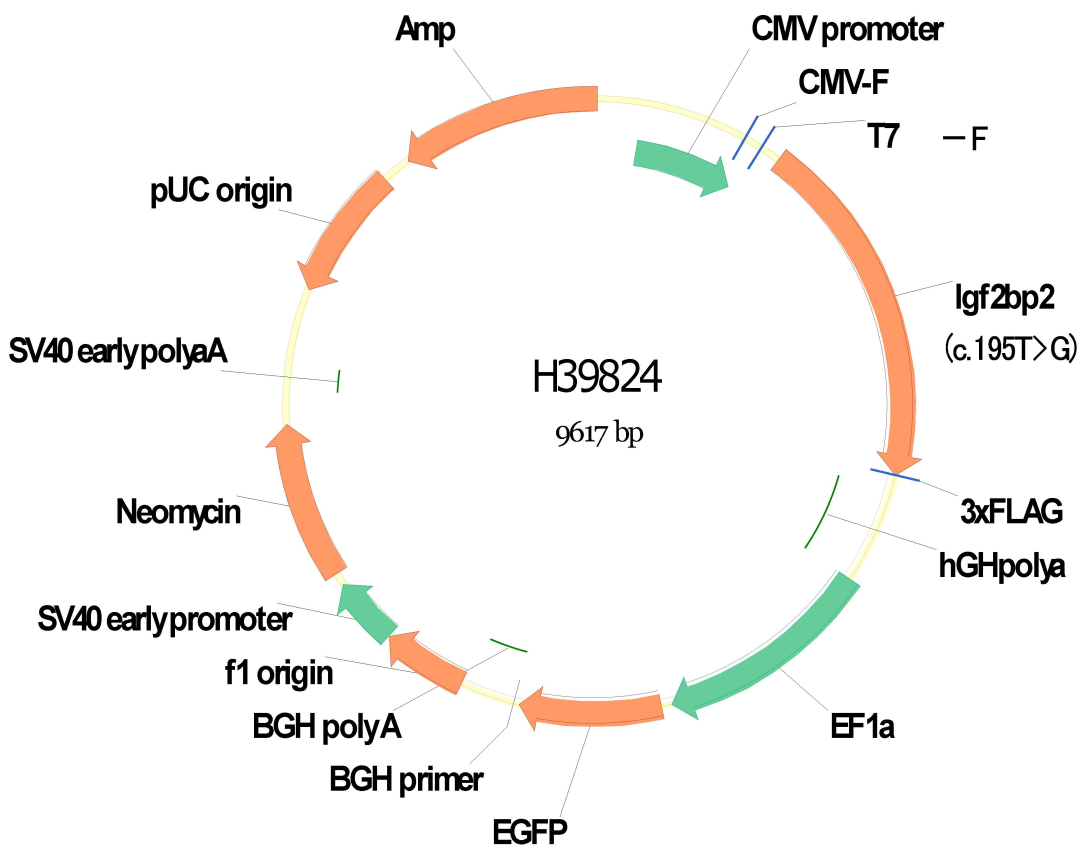

Supplemental Figure 14. Mutant *Igf2bp2* Plasmid Maps

Supplementary Table1. Antibody Information

Antibody name	Catalog NO.	Source	Manufacturer	Location
IGF2BP2/IMP2 polyclonal antibody	A1774	Rabbit	Abclonal	Wuhan, Hubei, China
GAPDH monoclonal antibody	60004-1-Ig	Mouse	proteintech	Wuhan, Hubei, China
RUNX2 polyclonal antibody	20700-1-AP	Rabbit	proteintech	Wuhan, Hubei, China
Osteopontin Polyclonal antibody	25715-1-AP	Rabbit	proteintech	Wuhan, Hubei, China
Osterix polyclonal antibody	DF7731	Rabbit	Affinity	Wuhan, Hubei, China
P53 Monoclonal antibody	60283-2-Ig	Mouse	proteintech	Wuhan, Hubei, China
p21 Polyclonal antibody	28248-1-AP	Rabbit	proteintech	Wuhan, Hubei, China
p16INK4a Polyclonal antibody	28416-1-AP	Rabbit	proteintech	Wuhan, Hubei, China
Acetyl-Histone H3 (Lys27) Recombinant antibody	82902-1-RR	Rabbit	proteintech	Wuhan, Hubei, China
Histone H3 Polyclonal antibody	17168-1-AP	Rabbit	proteintech	Wuhan, Hubei, China
DYKDDDDK tag Polyclonal antibody (Binds to FLAG® tag epitope)	20543-1-AP	Rabbit	proteintech	Wuhan, Hubei, China
HMGA1 Rabbit mAb	A4343	Rabbit	Abclonal	Wuhan, Hubei, China
Coralite488-conjugated Affinipure goat anti-rabbit IgG(H+L)	SA00013-2	Rabbit	proteintech	Wuhan, Hubei, China
Coralite594-conjugated Affinipure mouse anti-mouse IgG(H+L)	SA00013-3	Mouse	proteintech	Wuhan, Hubei, China
Goat anti-mouse IgG peroxidase-conjugated, H+L	BL001A	Goat anti-mouse	Biosharp	Heifei, Anhui, China
HRP-conjugated Affinipure goat anti-rabbit IgG(H+L)	SA00001-2	Goat anti-rabbit	proteintech	Wuhan, Hubei, China
Anti-Histone H3 (acetyl K27) antibody-ChIP Grade	ab4729	Rabbit	Abcam	Cambridge, UK
Phospho-Histone H2A.X (Ser139) (D7T2V) Mouse mAb	80312S	Mouse	Cell Signaling Technology	Massachusetts, United States
IgG	A7028	Mouse	Beyotime	Shanghai, China
IgG	A7016	Rabbit	Beyotime	Shanghai, China
IGF2BP2 Polyclonal antibody	11601-1-AP	Rabbit	proteintech	Wuhan, Hubei, China

Supplementary Table2. Primer Sequences for qRT-PCR

Gene	Forward Primer (5'-3')	Reverse Primer (5'-3')
Igf2bp2	CGCCAGACGAGAATGAGGAAGTG	GTATCTCTGCTCCTGCTGCTTCAC
Hmga1	CAGTGAAGTGCCAACTCCGAAG	TCCTCTTCCTCCTTCTCCAGTTTC
Gapdh	GTTGTCTCCTGCGACTTCA	TGGTCCAGGGTTTCTTACTCC
Tp53	TGAACCGCCGACCTATCCTTAC	GCACAAACACGAACCTCAAAGC
p16	CCGATTCAGGTGATGATGATGGG	CGGGCGGGAGAAGGTAGTG
Ocn	GCCGGAGTCTGTTCACTACC	GCGCTCTGTCTCTCTGACCT

Supplementary Table3. SiRNA Sequences

Sequence name	Sequences	Species
Negative control	Positive-sense strand: UUCUCCGAACGUGUCACGUTT<br>Antisense strand: ACGUGACACGUUCGGAGAATT	Mouse
Igf2bp2 siRNA	Positive-sense strand: UGACAAGAGAAGAGGCAAATT<br>Antisense strand: UUUGCCUCUUCUCUUGUCATT	Mouse
Hmga1 siR-#1	Positive-sense strand: GGAUGGGACUGAGAAGCGAGGTT<br>Antisense strand: CCUCGCUUCUCAGUCCCAUCCTT	Mouse
Hmga1 siR-#2	Positive-sense strand: CAAAGGGAAGCAAGAAUAAGGTT<br>Antisense strand: CCUUAUUCUUGCUUCCCUUUGTT	Mouse
Hmga1 siR-#3	Positive-sense strand: GCUCCAGGGAGGAAACCAAGGTT<br>Antisense strand: CCUUGGUUUCCUCCCUGGAGCTT	Mouse

Supplementary Table4. Probe Sequence of Fluorescence In Situ Hybridization

Probe Name	Probe sequence
Hmga1	TTCTGACTCCCGACCAGCGC

Supplementary Table5. The Top 10 Genes between RNA-seq and RIP-seq

Gene Symbol	logFC （RIP-seq）	P-Value	FDR	logFC （RNA-seq）	P-Value	FDR	Absolute Value （Product）
Hmga1	3.356	4.384E-46	2.465E-43	-2.759	5.314E-43	7.280E-40	9.259
Exo1	2.663	0.004	0.018	-2.118	7.216E-25	7.528E-24	5.640
Nr4a3	2.045	0.001	0.004	-2.596	9.577E-10	2.094E-09	5.309
Glrp1	2.663	0.004	0.019	-1.687	1.316E-07	2.430E-07	4.492
Ccdc74a	2.733	0.002	0.012	-1.523	8.630E-08	1.618E-07	4.162
Nes	1.427	0.008	0.031	-2.819	2.262E-41	1.549E-38	4.023
Ppargc1b	1.529	0.003	0.014	-2.585	2.431E-17	1.054E-16	3.952
Hmga2	1.128	5.408E-07	8.130E-06	-3.429	3.198E-43	4.818E-40	3.868
Gm13179	2.057	0.002	0.010	-1.861	5.855E-22	4.239E-21	3.828
Trpm6	2.590	0.006	0.027	-1.360	1.517E-05	2.381E-05	3.522

Supplementary Table7. Statistical Analysis of the Population-level Mutation Rate in IGF2BP2

	Age														Sex	
	<30	30-35	35-40	40-45	45-50	50-55	55-60	60-65	65-70	70-75	75-80	>80	Total(≤45)	Total(>45)	MALE	FEMEAL
Number of variant carriers	0	0	0	3	4	4	3	9	2	0	0	0	3	22	18	9
Number of individuals	9652	4669	5207	48016	63811	75306	86629	111640	86238	8247	4016	2388	67544	438275	799632	810304
Ratio(%)													0.004441549	0.005019679	0.002251035	0.001110694
